# Severe drug-associated anaphylaxis: a complementary descriptive analyses of registry cases and spontaneous reports

**DOI:** 10.1007/s00228-025-03868-w

**Published:** 2025-06-27

**Authors:** Patrick Christ, Diana Dubrall, Sabine Dölle-Bierke, Wojciech Francuzik, Matthias Schmid, Bernhardt Sachs, Margitta Worm

**Affiliations:** 1 Federal Institute for Drugs and Medical Devices (BfArM), Bonn, Germany; 2 Institute for Medical Biometry, Informatics and Epidemiology (IMBIE), University Hospital of Bonn, Bonn, Germany; 3 Division of Allergy and Immunology, Department of Dermatology, Venerology and Allergology, Charité - Universitätsmedizin Berlin, Corporate Member of Freie Universität Berlin, Humboldt-Universität Zu Berlin, Berlin Institute of Health, Berlin, Germany; 4 Department for Dermatology and Allergy, University Hospital RWTH Aachen, Aachen, Germany

**Keywords:** Adverse drug reactions, Allergy, Anaphylactic reaction, Drug-induced anaphylaxis, Pharmacovigilance study

## Abstract

**Purpose:**

Drugs are among the most common triggers of severe anaphylactic reactions in adults. The aim of our study was to identify the most frequently suspected drugs and associated factors of severe drug-associated anaphylactic reactions in two different data sources. Moreover, the impact of the route of administration (oral versus intravenous) was investigated.

**Methods:**

Severe drug-associated anaphylactic reactions from Germany were analysed in 1046 cases of the European Anaphylaxis Registry and in 1878 spontaneous reports of the European adverse drug reaction (ADR) database EudraVigilance.

**Results:**

Several analgesics, antibiotics and contrast media were among others reported most frequently in both data sources. In addition, antineoplastic and immunomodulating agents were commonly reported in spontaneous reports. As associated factors, thyroid disorders, asthma and chronic obstructive pulmonary diseases, as well as cardiovascular diseases, were frequently reported in both. Serious reactions such as cardiac arrest were more commonly reported for intravenously administered drugs, while skin reactions were more common for orally administered drugs.

**Conclusions:**

The analyses of two datasets differing regarding their data collection enables to get a more complete picture of severe anaphylactic reactions in real world settings. Our study confirms that patients with thyroid disorders, cardiovascular and respiratory diseases (e.g. asthma) might carry a higher risk to develop severe anaphylactic reaction. The more serious course of anaphylactic reactions related to intravenously compared to orally applied drugs may result from the faster availability of intravenously administered drugs or differences among the patient populations.

**Supplementary Information:**

The online version contains supplementary material available at 10.1007/s00228-025-03868-w.

## Purpose

An anaphylactic reaction is a severe, potentially life-threatening systemic hypersensitivity reaction, induced by specific immunoglobulin E (IgE) or non-IgE dependent mechanisms [1–3]. As a result, symptoms occur in different organ systems affecting the skin, lungs, cardiovascular system and gastrointestinal tract [4].

Anaphylactic reactions in adults are most frequently induced by insect venoms, drugs and food [4–10]. Compared to the other causes, drug-associated anaphylactic reactions in adults are more often severe and more likely to result in a fatal outcome [11, 12].

The aim of our study was to investigate severe drug-associated anaphylactic reactions regarding patient demographics, suspected drugs, clinical symptoms and associated factors in two different data sources, the European Anaphylaxis Registry and the European adverse drug reaction (ADR) database EudraVigilance. We chose this combined approach to obtain a more complete picture as the databases collect different information and may include different patient populations. Furthermore, additional EudraVigilance analyses were carried out to determine whether the symptoms of anaphylactic reactions differed depending on the route of administration (oral versus intravenous).

## Methods

The term anaphylactic reaction is superordinately used throughout this manuscript as a synonym for the terms anaphylaxis and anaphylactoid reactions.

### EudraVigilance

The European Medicines Agency’s (EMA) adverse drug reaction (ADR) database EudraVigilance contains all spontaneous reports of suspected ADRs from all countries of the European Economic Area. In EudraVigilance, active substances are coded according to the “EudraVigilance medicinal product dictionary” and ADRs are coded according to the MedDRA terminology, which consists of five hierarchical levels [13, 14]. The “Preferred term” (PT) level of the MedDRA terminology describes, among others, symptoms, diagnoses and laboratory results. “Standardized MedDRA Queries” (SMQ) are established and validated collections of specified PTs that can be used to identify all ADR reports related to specific diagnoses or symptom(-groups) [15].

A spontaneous report is classified as serious if any of the reported ADRs were life-threatening or fatal, resulted in a hospitalization or prolongation thereof, permanent injuries or impairments, abnormalities or medical and surgical interventions [16]. Note that the seriousness according to the legal definition does not necessarily correspond to its clinical severity.

Further information on reporting channels and the definition of ADRs is described elsewhere [17].

### The European Anaphylaxis Registry

More than one hundred allergy centres from ten European countries and Brazil report anaphylactic reactions to the Anaphylaxis Registry. After obtaining written consent from the patients affected, the study centres gather information via a standardized online questionnaire. Trained specialists enter pseudonymized data from patients who have experienced a severe anaphylactic reaction within the last 12 months at the time of admission to the allergy centre via this questionnaire, which comprises data to record the demographics of the patient (age, sex), suspected triggers, symptoms, associated factors, treatment of the anaphylactic reaction and preventive measures [18].

### Identification of cases/reports in both data sources

Suitable criteria were defined for each data source considering database-specific criteria to create inclusion and exclusion criteria as similar as possible.

We cannot exclude that single cases from the Anaphylaxis Registry were also reported to EudraVigilance and are therefore included in both datasets.

#### Identification of spontaneous ADR reports in EudraVigilance

**Fig. 1:**
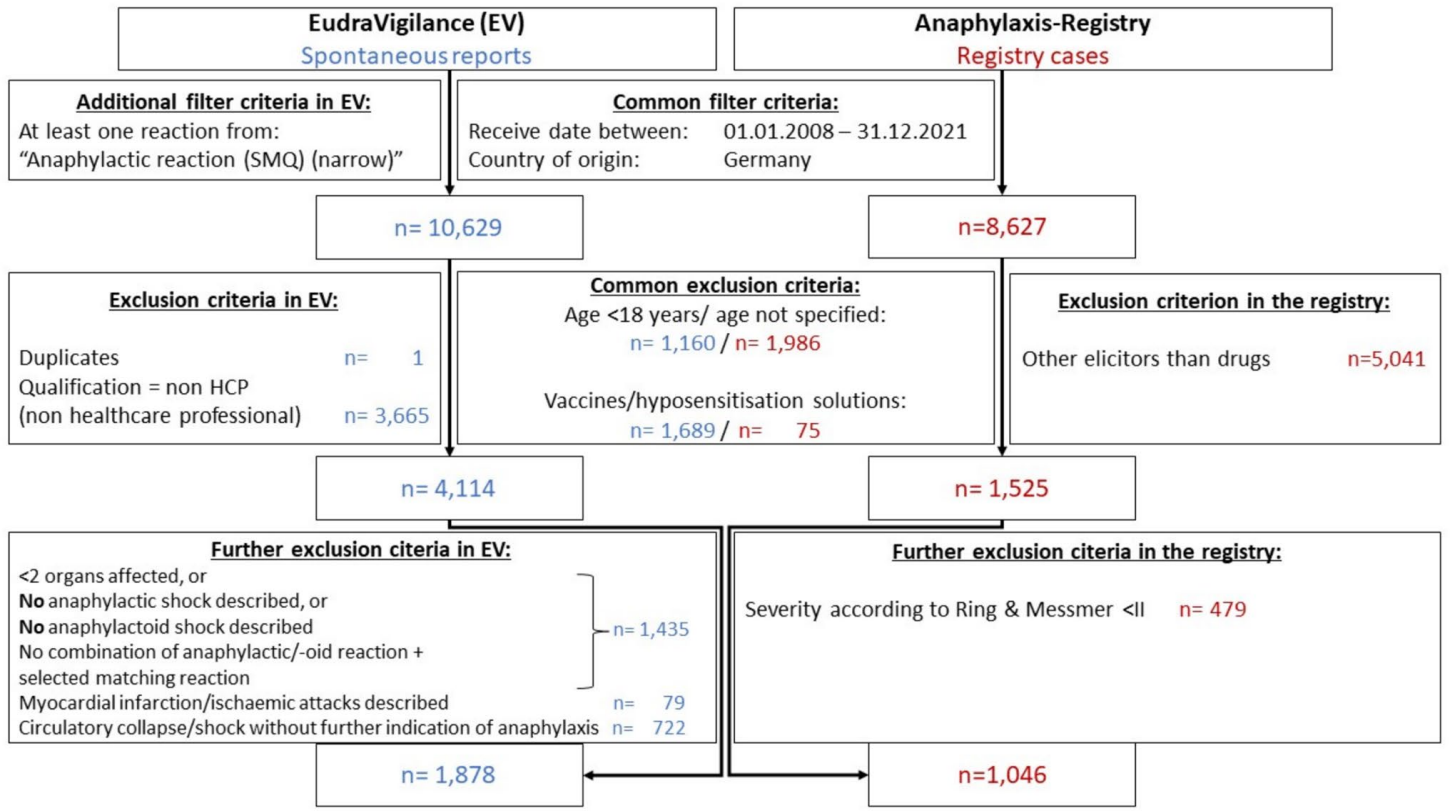
Flowchart: identification of severe anaphylactic reactions in the spontaneous reports and the registry cases. Legend Fig. 1: EV = EudraVigilance; registry = Anaphylaxis Registry; SMQ = Standardised *MedDRA* Queries includes defined and validated PT of MedDRA terminology describing an anaphylactic reaction [19]; non-HCP = non-Health Care Professionals corresponds to all reporters without a qualification as healthcare professional, for example patients, their relatives, or lawyers

All spontaneous reports from Germany, received between 01.01.2008 and 31.12.2021, reported by a healthcare professional (e.g. physician, pharmacist) referring to adults (≥ 18 years) including at least one reaction of the SMQ “Anaphylactic reaction (narrow)” were extracted from EudraVigilance [15]. Reports mentioning at least one vaccine or hyposensitization solution as suspected/interacting drug were excluded from further analysis (Fig. 1). Furthermore, all reports describing type I anaphylactic reactions (exclusively involving skin reactions) according to the Ring and Messmer classification were excluded [19]. In summary, all ADR reports describing symptoms of at least two of the organ systems skin, gastrointestinal tract, respiratory and cardiovascular system and/or in which the PT *anaphylactic shock* or *anaphylactic reaction* was coded were considered.

A random sample of 205 ADR reports was individually assessed by the authors PC, DD and BS regarding their (i) causal relationship according to WHO criteria between the drug reported as suspected/interacting and the occurrence of the anaphylactic reaction [20] and (ii) the accuracy of the diagnosis of the anaphylactic reaction. To harmonize the evaluation, ten ADR reports were randomly selected and initially assessed by each assessor. The agreement of evaluation was determined by the calculation of Fleiss’ kappa [21]. Based on the individual assessment, we recognized that certain combinations of symptoms not exclusively described anaphylactic reactions. This was particularly observed for ADR reports coded with the PT’s circulatory collapse and shock. Further evaluations of these reports showed that only those reports including these PTs and additionally describing symptoms of the respiratory and cardiovascular systems reliably characterised anaphylactic reactions. Any other combinations of symptoms rather described other diseases and, thus, were excluded from further analysis. Duplicates identified during the assessment were removed from the dataset. The final spontaneous report dataset included 1878 reports of serious anaphylactic reactions and 119 (6.3%) of the individual assessed reports were contained. Within these reports, an anaphylactic reaction was distinctly evident in 99 reports (83.2%). In five ADR reports (4.2%), an anaphylactic reaction was not apparent, and in 15 reports (12.6%) the assessment was not possible (see Supplementary Information Table 2). Regarding the causal relationship, 90 reports (76.5%) were classified as at least possible (possible = 50 reports, probable = 36 reports, certain = four reports), five reports (4.2%) as unlikely, and 24 reports (20.2%) as not assessable. Regarding the ten reports assessed by each assessor, the calculated Fleiss’ kappa value of 0.8 (0.5–1.0) implies as a substantial agreement according to Landis and Koch [21].

#### Identification of cases in the Anaphylaxis Registry

All German cases included in the Anaphylaxis Registry, reported between 01.01.2008 and 31.12.2021 with a severity of at least grade II according to Ring and Messmer for adults (≥ 18 years) were analysed [19]. Cases in which vaccines or hyposensitization solutions were specified as causative agents were excluded from further analysis. In the anaphylaxis registry, some of the suspected drugs or reported associated factors or diseases are assigned to higher-level drug groups or diagnoses in the questionnaire.

A total of 73 cases, 45 of them in 2014 related to local anaesthetics, were exclusively reported from one study centre. This resulted in an overall higher number of cases in 2014, and the total number of reports from this centre accounts for approximately half of all cases relating to local anaesthetics.

### Ethics

The presented study is a retrospective analysis of pseudonymised ADR reports from EudraVigilance. According to the local ethics committee of the Medical Faculty of Bonn, no ethics approval is needed for this study (file no. 458/20 and 100/21).

The German anaphylaxis register was approved by the ethics committee at Charité-Universitätsmedizin Berlin, Germany (EA1/079/06), accredited by the local ethics committees in the participating centers, and is registered on ClinicalTrials.gov (Identifier: NCT05210543).

### Descriptive analyses

Both datasets were descriptively analysed regarding the number of reports per year, the number of reports classified as life-threatening, fatal, or leading to hospitalization (or prolongation thereof), the age and sex of the patients, the symptoms, associated factors, co-medications and suspected/interacting drugs reported. Additionally, in the registry, the number of specifically severe cases was determined by analysing the number of cases assigned as grade IV anaphylactic reactions according to Ring and Messmer [19].

In the spontaneous reports, the most reported drugs were grouped to higher-level drug groups and the reported symptoms were assigned to their organ systems to increase comparability of results of the two databases.

The term associated factors relates to the medical history (corresponds largely to concomitant diseases) in the spontaneous reports, whereas a selection of concomitant diseases and accompanying circumstances (e.g. physical exercise) are covered in the registry cases. The percentual shares in the analyses of associated factors and co-medications are based on the total number of reports, which included at least one associated condition or co-medication.

Note that a sub differentiation, e.g. of certain concomitant diseases (e.g. asthma/COPD) or medications (e.g. quinolone antibiotics) was not possible for cases from the Anaphylaxis Registry.

#### Additional descriptive analysis of spontaneous reports

The route(s) of application of each suspected drug was determined in the spontaneous reports.

All spontaneous reports with an allergic or hypersensitivity reaction reported in the patient’s history were identified by the application of the HLGT “allergic conditions”. Further on, reports having a higher probability of describing an allergy or hypersensitivity to drugs were identified by appropriate PT of the HLGT “allergic conditions” (*n* = 157). An individual case assessment of these reports (*n* = 157) was performed to further characterize if a pre-existing allergy or hypersensitivity reaction to a drug was reported and to determine if the already known allergy against the drug relates to the drug reported as suspected.

### Statistical analyses

The mean value (mean) together with the standard deviation (± sd) and the median (Md) together with the first and third quantiles were calculated for the number of reports/cases per year and patient’s age.

To identify characteristics more frequently reported in reports/cases identified for a specific drug group, odds ratios (OR) according to the principles of reporting OR together with their 95% confidence intervals (95% CI) compared to all remaining reports/cases, including all other drugs, were calculated [22]. The drug groups were compared regarding the categories of sex and age group of the patients, medical histories, associated factors, co-medications, number and type of affected organ systems and the criterion of hospitalization.

In all OR calculations, it was assumed that a value was more likely to be present in the group under consideration if the lower CI was greater than 1.0 and less likely if the lower CI was smaller than 1.0. To restrict the analysis to the most important categories, at least ten reports had to be present for the respective specification.

#### Additional statistical analysis of spontaneous ADR reports

To identify characteristics more frequently reported in spontaneous reports in which the drug(s) was/were exclusively administered orally (*n* = 371) or intravenously (*n* = 811), ORs with their 95% CIs were calculated. Thereby, the sex and age group of the patients, associated factors, reported symptoms and the occurrence of death were considered. A more frequent occurrence for reports exclusively including orally applied drugs was assumed if the lower CI was greater than 1.0 and a less frequent occurrence for orally applied drugs but a more frequent occurrence for intravenously applied drugs was assumed if the upper CI was lower than 1.0. To restrict the analysis to the most important categories, at least 15 reports had to be present for the respective specification.

## Results

### Spontaneous reports

#### Characteristics of spontaneous reports

Throughout the study period (2008–2021), the number of spontaneous reports decreased from 2008 to 2021 (− 69.3%; mean = 134.1 ± 36.5 reports/year)) (Supplementary Information Table 1).

**Fig. 2:**
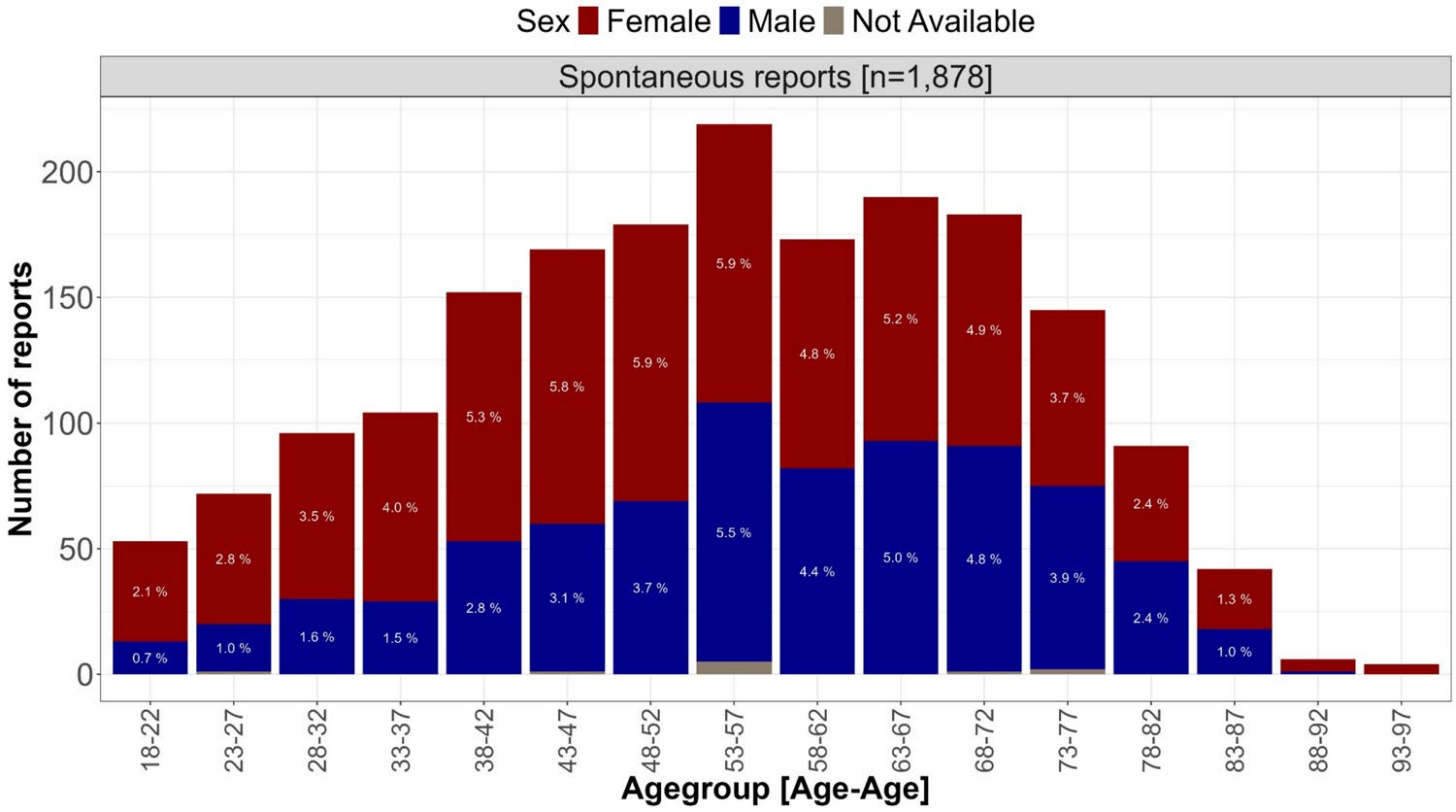
Distribution of age and sex of the patients in the spontaneous reports. Figure 2 visualises the absolute number of patients of the respective age groups in the spontaneous reports. Colours indicate the proportion of reports on females (red), males (blue) and unknown sex (grey). The shares of the sexes and age groups in the total dataset of spontaneous reports are shown in each bar using a white font colour

In median patients in spontaneous reports were 55 [IQR 42–68] years old and 58.1% of them were females (Fig. 2).

Related to the number of reports with respective information, 55.6% of the spontaneous reports described a hospitalization or prolongation thereof, 8.5% a fatal outcome and 55.4% a life-threatening situation.

#### Associated factors

At least one associated factor and one co-medication was observed in 75.4% and 42.0% of the spontaneous reports, respectively (Supplementary Information Table 2).

Among these reports, various atopy-associated diseases, such as *asthma* (6.9%), *seasonal allergy* (5.9%) and *food allergy* (3.3%) were identified in the 20 most frequently reported medical histories (Supplementary Information Table 2). *Drug hypersensitivity* was reported as the second most common associated factor overall (8.8%). In summary, 434 (23.1%) spontaneous reports described a known *allergy or hypersensitivity* reaction in the patient’s history (Supplementary Information Table 3). Of the 157 spontaneous reports individually analysed for a pre-existing allergy/hypersensitivity reaction to a drug, 9.0% reported an already known allergy to the reported suspected drug (whole dataset: 0.7% (12/1878)) (Supplementary Information Table 3). In the remaining reports, allergies to other drugs or other triggers (e.g. food) were described.

Considering the relevant drug groups of co-medications within the ADR reports with respective information, non-steroidal anti-inflammatory drugs (NSAIDs) were reported in 18.7%, beta blockers in 16.4%, proton pump inhibitors (PPI) in 12.7%, thyroid therapeutics in 12.4% and angiotensin-converting enzyme inhibitors (ACEi) in 12.1% of the spontaneous reports.

#### Symptoms of anaphylactic reactions

Considering the superordinate organ systems, in 86.7% of the reports symptoms of the cardiovascular system (e.g. *circulatory collapse* 10.6%), in 45.4% involvement of the skin (e.g. *urticaria* 11.4%), in 43.7% symptoms of the respiratory system (e.g. *dyspnoea* 28.3%) and in 24.7% symptoms of the gastrointestinal tract (e.g. *nausea* 11.9%) were reported. Note that, regarding the symptom level analysis, the overarching diagnoses *anaphylactic shock* (56.9%) and *anaphylactic reaction* (31.4%) were most frequently reported and were assigned to the cardiovascular system (Supplementary Information Table 2).

#### Suspected drugs and drug groups

In 22.2% of the spontaneous reports, more than one suspected/interacting drug was reported. The most frequently reported drug was moxifloxacin (5.0%), followed by human immunoglobulin (4.6%), cefuroxime (4.0%), ferric carboxymaltose/polymaltose (3.6%) and metamizole (3.4%) (Supplementary Information Table 2).

**Fig. 3:**
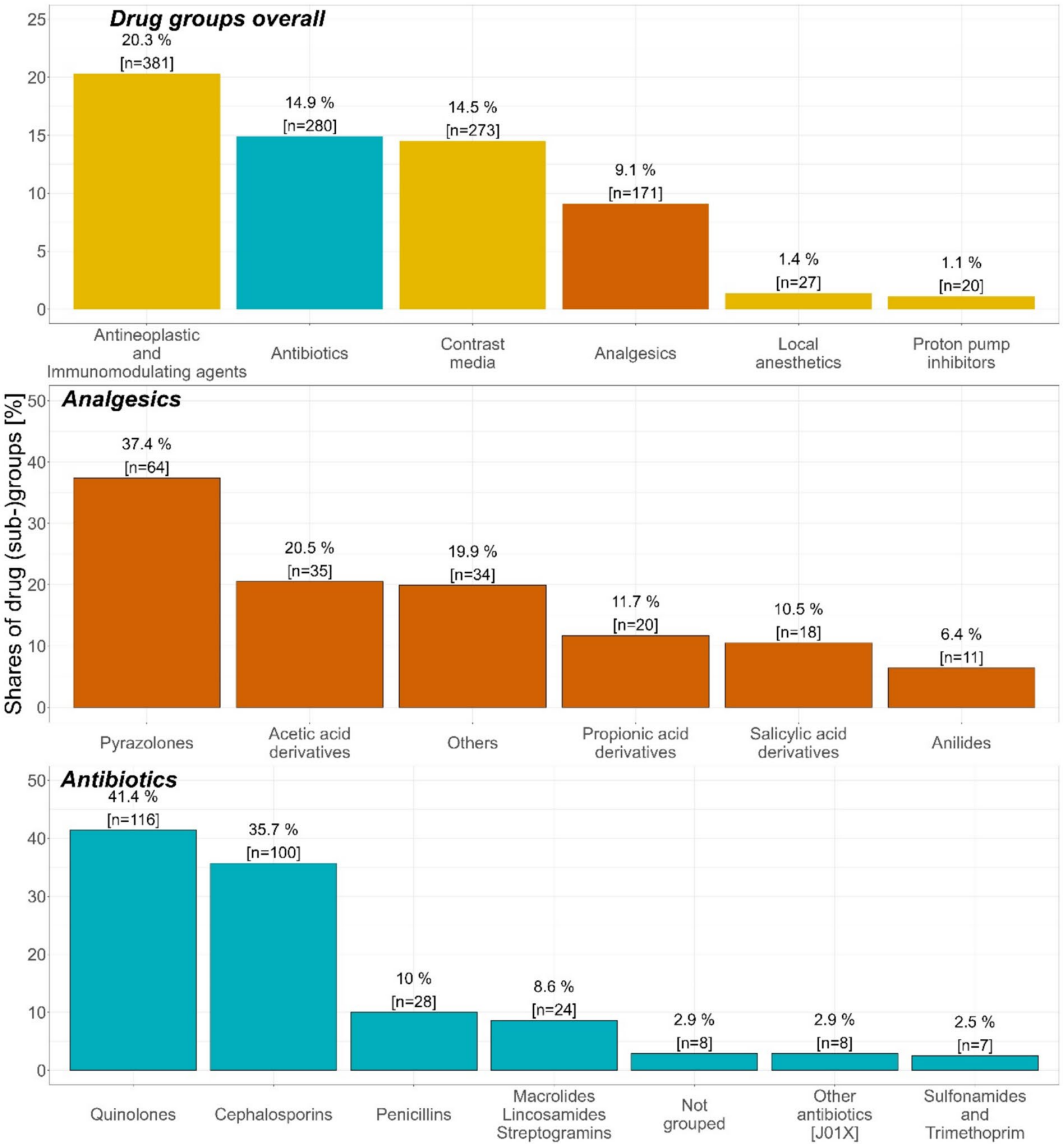
Analysis of the drug groups of interest in the spontaneous reports. Figure 3 shows the number of spontaneous reports and their proportion of the total number of spontaneous reports for selected drug groups of interest. A more in-depth overview regarding the distribution of the (sub)groups for analgesics and antibiotics is also shown. J01X: ATC group of “Other antibiotics”, e.g. fosfomycin

On the aggregated drug class level, antineoplastic and immunomodulating agents (20.3%) were the drug class most frequently reported as suspected, followed by antibiotics (14.9%) and contrast media (14.5%) (Fig. 3).

#### Characteristics more frequently reported in spontaneous reports of anaphylactic reactions to individual drug groups

Compared to the respective reports of all other drug groups (Supplementary Information Table 4), among others, the following characteristics were more frequently reported in the following:

- Analgesics: asthma (OR 1.9 [1.1–3.3]) and hypersensitivity (OR 2.1 [1.2–3.7]) as associated factors and swollen tongue as ADR (OR 2.2 [1.1–4.3]),
- Antibiotics: bronchitis (OR 28.0 [8.0–98.0]), *obesity* (OR 2.0 [1.1–3.4]), *tobacco abuse* (OR 2.8 [1.6–4.9]) and *tobacco user* (OR 2.5 [1.5–4.1]) as associated conditions, patient’s age of 45–64 years (1.6 [1.2–2.0]),
- Contrast media: coronary artery diseases (OR 2.1 [1.3–3.3]) as associated condition, male sex (OR 1.5 [1.1–1.9]),
- Local anaesthetics: one organ system affected (OR 2.5 [1.1–5.3]), *anaphylactic shock* (OR 2.7 [1.1–6.7]) as ADR, female sex (OR 3.2 [1.2–8.5]),
- Antineoplastic and immunomodulating agents: autoimmune diseases (*Crohn’s disease* OR 6.4 [3.4–11.9]; *multiple sclerosis* OR 11.6 [5.4–25.1]; rheumatoid arthritis OR 5.4 [2.2–12.9]) as associated conditions,
- PPI: patient’s age of 45–64 years (OR 2.9 [1.2–7.4]).

#### Characteristics more frequently reported in spontaneous reports of orally or intravenously administered drugs

In 76.3% of all spontaneous reports, the route of administration was reported for at least one drug, of which 43.2% (*n* = 811) exclusively involved intravenously and 19.8% (*n* = 371) exclusively orally administered drugs (Supplementary Information Table 2).

**Fig. 4:**
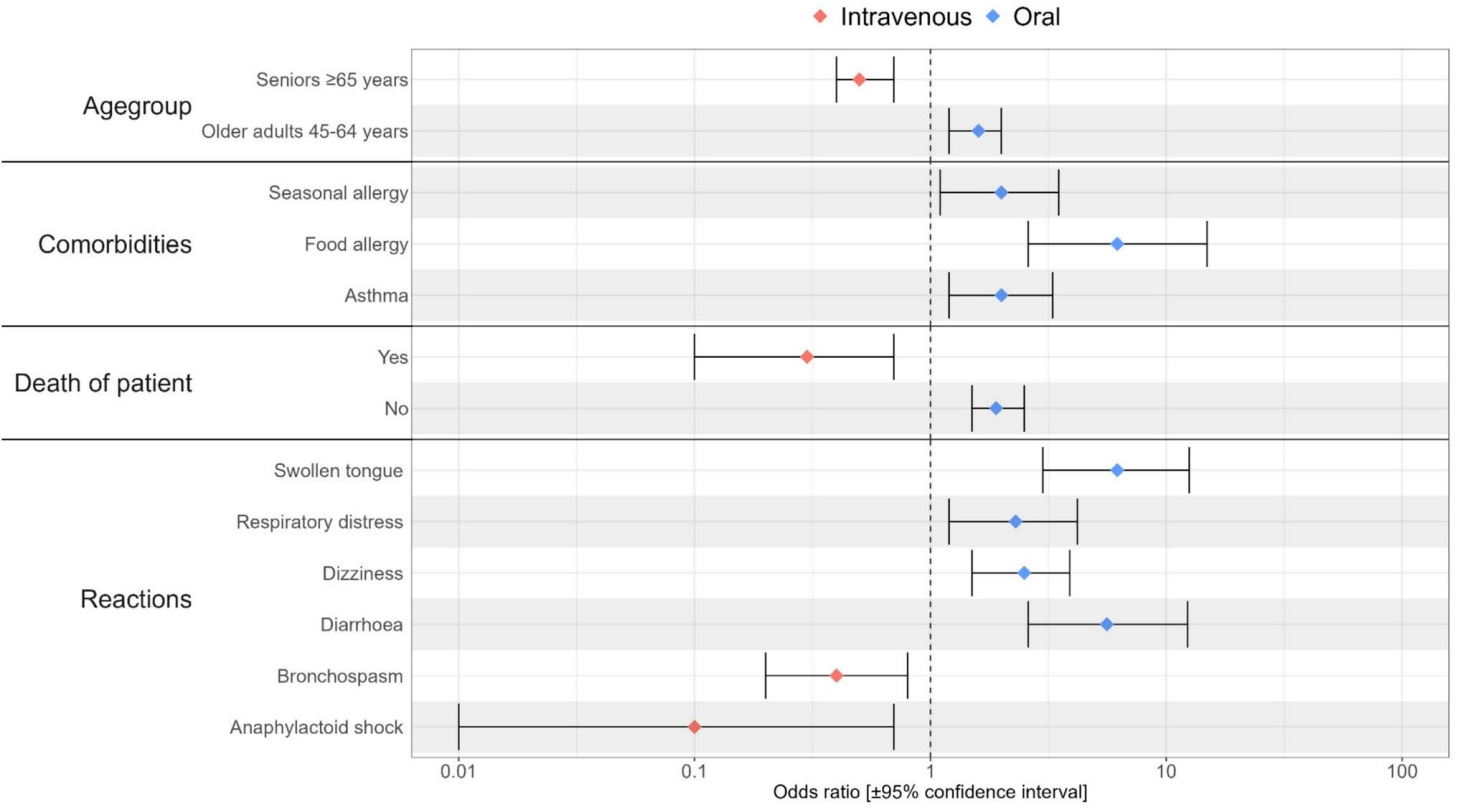
Comparative analysis of the spontaneous reports with exclusively orally or intravenously administered drugs (selection). Figure 4 shows the reporting odds ratios with associated ± 95% confidence intervals (illustrated by error bars) of a selection of factors identified in the categories age group, comorbidities, death and reactions more frequently reported in the spontaneous reports exclusively involving oral versus intravenous applied drugs *(for all results of this evaluation please refer to table appendix 5).* A more frequent occurrence in the spontaneous reports with exclusively orally applied drugs was assumed if the lower CI was greater than 1.0 and a more frequent occurrence in the spontaneous reports with exclusively intravenously applied drugs was assumed if the upper CI was lower than 1.0. Odds ratios indicating a more frequent occurrence with intravenously administered drugs are highlighted by red point estimators and those indicating a more frequent occurrence for orally administered drugs by blue point estimators

Compared to reports of exclusively containing orally applied drugs, the death of the patient (*n* = 50) (OR 0.3 [0.1–0.7]), patient’s age of 65 years or older (OR 0.5 [0.4–0.7], *Crohn's disease* (OR 0.1 [< 0.1–0.6]), *renal failure* (OR 0.2 [0.1–0.7]) and *chemotherapy* (OR 0.1 [< 0.1–0.9]) as associated conditions and *anaphylactoid shock* (OR 0.1 [< 0.1–0.7]), *cardiac arrest* (OR 0.2 [0.1–0.5]) and *bronchospasm* (OR 0.4 [0.2–0.8]) as ADRs were more frequently reported in reports exclusively containing intravenously applied drugs (Fig. 4, Supplementary Information Table 5).

In contrast, patient’s age of 45 and 64 years (OR 1.6 [1.2–2.0]), *food allergy* (OR 6.2 [2.6–14.9]), *asthma* (OR 2.0 [1.2–3.3]), and *seasonal allergy* (OR 2.0 [1.1–3.5]) as associated conditions, skin reactions, swellings (e.g*. swollen tongue* (OR 6.2 [3.0–12.5])), *dizziness* (OR 2.5 [1.5–3.9]), *respiratory distress* (OR 2.3 [1.2–4.2]) and *diarrhoea* (OR 5.6 [2.6–12.3]) as ADRs were more commonly reported for reports of exclusively orally compared to those with exclusively intravenously applied drugs.

### Registry cases

#### Characteristics of registry cases

The number of registry cases (mean = 74.7 ± 62.6 cases/year) remained almost equal from 2015 onwards (Supplementary Information Table 6).

**Fig. 5:**
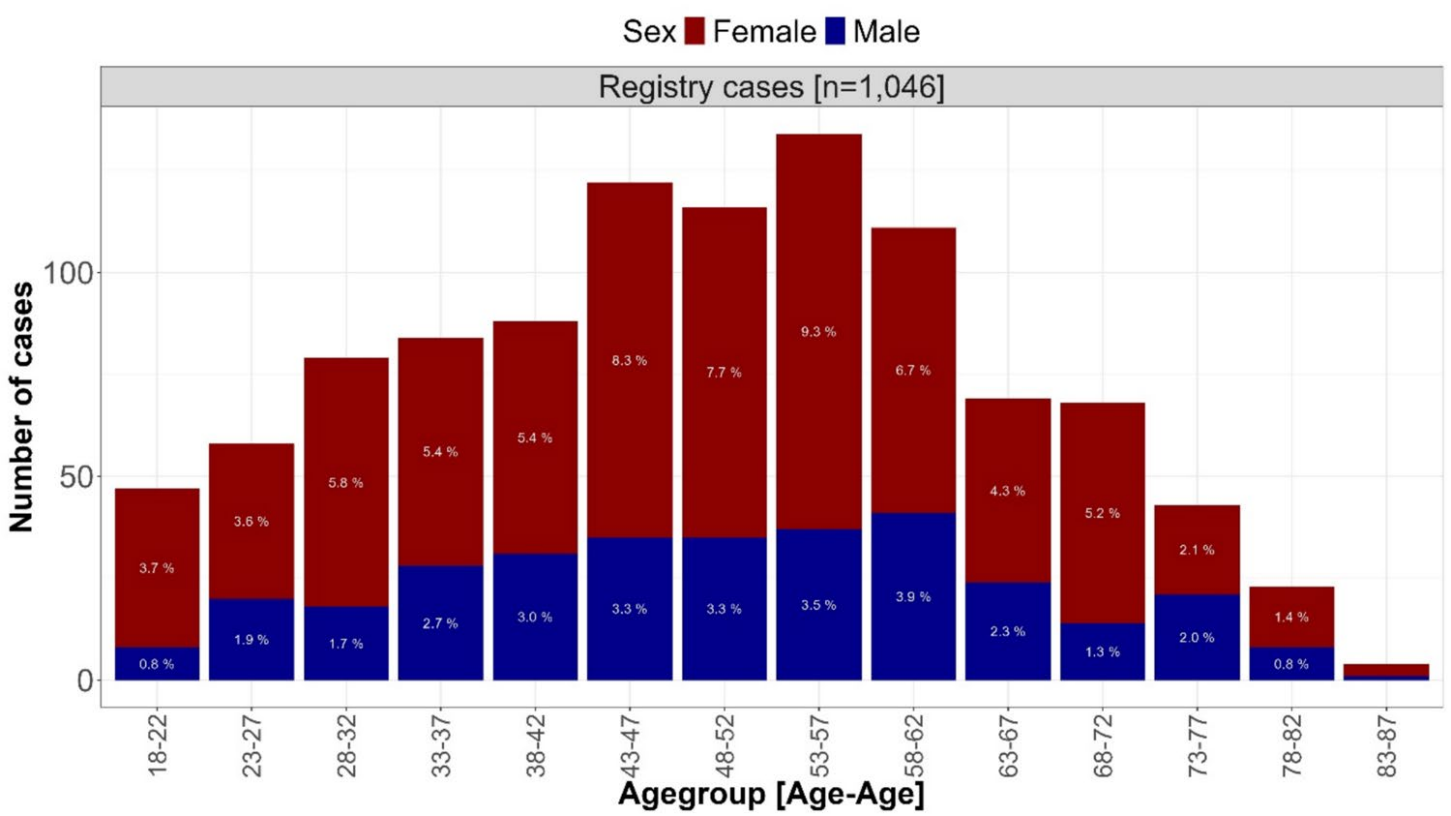
Distribution of age and sex of the patients in the registry cases. Figure 5 visualises the absolute number of patients of the respective age groups in the registry cases. Colours indicate the proportion of reports on females (red), males (blue) and unknown sex (grey). The shares of the sexes and age groups in the total dataset of registry cases are shown in each bar using a white font colour

In median, the patients in the registry cases were 49 [37–60] years old, and 69.3% of them were females (Fig. 5, Supplementary Information Table 6).

A hospitalization was reported in 26.9% and a fatal outcome in 1.0% of the registry cases with respective information. According to Ring and Messmer, in 6.9% of the registry cases, the anaphylactic reaction was classified as grade IV.

#### Associated factors

Besides cardiovascular diseases (36.7%) and asthma/COPD (20.8%), different atopy-associated factors such as allergic rhinitis/rhinoconjunctivitis (19.9%) and atopic dermatitis/eczema (5.1%) were among the most frequently reported conditions in the registry cases with information (Supplementary Information Table 7).

Beta blockers were reported in 25.1%, thyroid therapeutics in 21.0%, ACEi in 17.2% and PPI in 14.4% of the registry cases in the superordinate analyses of relevant drug groups within the co-medication in reports with information.

#### Symptoms of anaphylactic reactions

In 76.9% of the cases, the skin (e.g. angioedema 39.8%), in 71.6% the cardiovascular system (e.g. hypotension/collapse 37.2%), in 71.2% the respiratory tract (e.g. dyspnoea 59.8%) and in 29.8% the gastrointestinal tract (e.g. nausea 19.2%) was involved (Supplementary Information Table 7).

#### Suspected drugs and drug groups

**Fig. 6:**
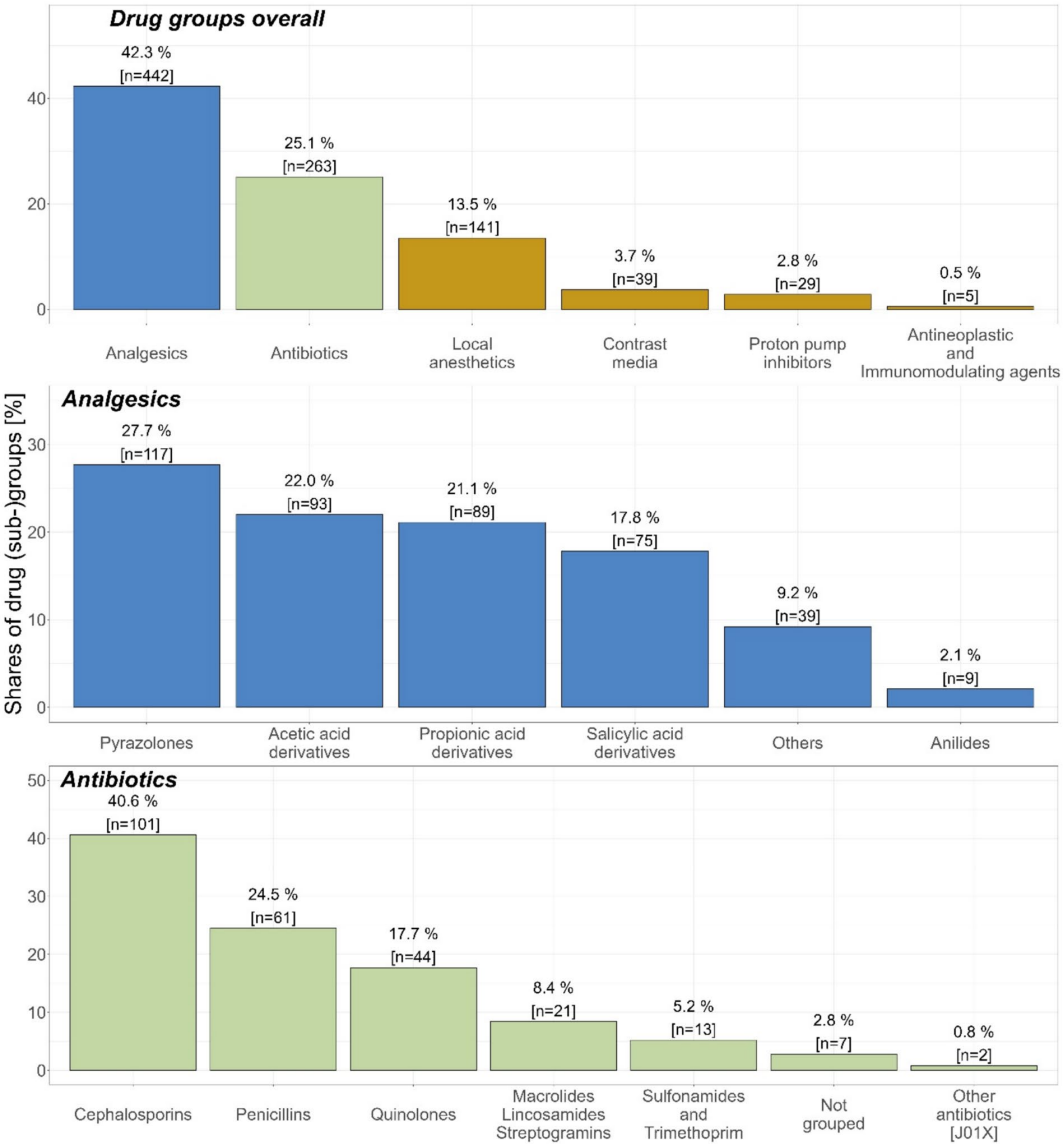
Analysis of the drug groups of interest in the registry cases. Figure 6 shows the number of the registry cases and their proportion on the total number of registry cases for selected drug groups. A more in-depth overview regarding the distribution of the (sub)groups for analgesics and antibiotics is also shown. J01X: ATC group of “Other antibiotics “, e.g. fosfomycin

The most frequently reported drugs in the registry cases were metamizole (11.8%), cephalosporins (10.4%), diclofenac (9.4%), ibuprofen (9.0%) and acetylsalicylic acid (7.2%) (Supplementary Information Table 6). On the superordinate drug class level most of the cases reported drugs belonging to analgesics (42.3%), followed by antibiotics (25.1%), local anaesthetics (13.5%), contrast media (3.7%) and narcotics (3.7%) (Fig. 6).

#### Characteristics more frequently reported in registry cases of anaphylactic reactions to individual drug groups

Compared to the respective reports of all other drug groups (Supplementary Information Table 8), among others, the following characteristics were more frequently reported in the following:

- Analgesics: asthma (OR 1.6 [1.1–2.2]) and exercise moderate (OR 2.4 [1.5–3.8]) as associated factors, angioedema (OR 1.8 [1.4–2.3]) as ADR, male sex (OR 1.6 [1.2–2.1]),
- Antibiotics: infection (OR 6.7 [3.7–10.0]) as associated condition, female sex (OR 1.7 [1.2–2.3])
- Contrast media: cardiovascular disease (OR 2.4 [1.2–4.6]) as associated condition.
- Local anaesthetics: only one organ system affected (OR 4.0 [2.3–7.0], loss of consciousness (OR 1.7 [1.1–2.6]) as ADR, stress likely (OR 1.9 [1.2–3.1]) as associated condition.

## Discussion

This analysis of severe drug-associated anaphylactic reactions in Germany represents, to the best of our knowledge, the first complementary analysis of this magnitude investigating two different data sources. The total numbers of cases/reports, especially those leading to hospitalisation, highlight the enormous relevance of this subject [23–25].

### Differences between the datasets

Both data sources differ regarding their collection of data and may, thus, represent different patient populations. At first, the cases from the anaphylaxis registry are reported by physicians of specialised allergy centres on a voluntary basis, while the spontaneous reports of anaphylactic reactions may be reported by any healthcare professionals (e.g. physicians and pharmacists) in Germany who are obliged by their professional codes of conduct to report ADRs. For the spontaneous ADR reports, reporting bias cannot be excluded. According to a German study, physicians spontaneously report severe ADRs and ADRs related to new drugs more often than non-serious ADRs or those related to well-known drugs [26]. Thus, serious anaphylactic reactions and those related to new drugs may be more often observed in the spontaneous reports from EudraVigilance than in the anaphylaxis registry. Further on, we observed a substantially lower number of spontaneous ADR reports describing anaphylactic reactions in 2021. A decrease of the overall (i.e. irrespective of the ADR) number of spontaneous ADR reports was also observed in another study of our research group [27]. Both observations might be related to a preferential reporting of ADRs related to COVID-10-vaccines compared to other drugs as assumed by others in the respective period [28]. Further on, different types of information are collected by reporting an ADR spontaneously, e.g. via the online platform to EudraVigilance or by reporting an anaphylactic reaction to the anaphylaxis registry by using a specific questionnaire. As an example, in our study information about the route of administration could only be analysed in EudraVigilance and information about physical activity only in registry cases. Since very mild courses of anaphylactic reactions, only involving the skin, may be underrepresented in the anaphylaxis registry, we decided to exclude grade I anaphylactic reactions according to the classification of Ring and Messmer [19]. The classification of anaphylactic reactions according to their severity by Ring and Messmer [19] is not included in the spontaneous reports. For this reason, we tried to re-create a dataset of spontaneous reports corresponding to grade II, III and IV anaphylactic reactions by Ring and Messmer [19] by using specific MedDRA terms or combinations of them and by excluding ADR reports exclusively describing skin or single organ involvement. The aim was to generate two datasets as similar as possible regarding their inclusion criteria. All the differences mentioned above regarding the collection of data, or the generation of the datasets may have impacted on the differences of the results between the datasets to an unknown extent. However, in our opinion, this complementary approach considering two different data sources probably reflecting different types of patient populations allows us to obtain a more complete picture of anaphylactic reactions in real-world settings. This is one of the greatest strengths of our analysis.

### Patient characteristics

In other studies, females and middle-aged patients were more likely to experience anaphylactic reactions as also observed in our study [4, 24, 29–33]. This finding may be attributed to sex and age-specific differences considering the intake of specific drugs and diseases. As an example, in a German study analgesic use was higher for females than for males and was higher for middle-aged patients than patients older than 65 years [34]. Among others, a higher use of anti-infectives, antineoplastic and immunomodulating agents, drugs of the category varia (including contrast media) and drugs of the category alimentary tract and metabolism (including PPI) for females than for males was observed [35], too. All the aforementioned drugs/drug classes were frequently reported in both datasets of our analysis. The impact of age has already been discussed in literature assuming a potential association between the more frequent intake of drugs and the higher proportion of polypharmacy in older than in younger patients [36]. Further on, elevated cardiovascular vulnerability increases with increasing age and could be associated with a higher risk to develop (severe) anaphylactic reactions, too [24, 36]..

### Relevant drugs and drug groups

Analgesics and antibiotics were, consistent with other studies, among the most commonly suspected drugs of severe anaphylactic reactions in both data sources [25]. This could, however, also reflect the frequent use of these drug groups [4, 32, 37].

The number of cases related to local anaesthetics in the registry (*n* = 141) was influenced by the higher number of reports (*n* = 45, 31.9%) received from one centre in 2014. Exclusion of these cases was considered, but no impact on the results presented was observed [7], which is why we left the cases in our dataset. Nevertheless, overreporting in the registry or underreporting in the spontaneous reports has to be considered [26, 38].

As in our analysis of spontaneous reports, antineoplastic and immunomodulating agents and contrast media were also identified as the most frequently reported suspected drugs for anaphylactic reactions in analyses of the Japanese and the American ADR databases (FEARS and JADER). [29, 39].

### Relevant co-medications and medical histories

Some of the co-medications described in the literature to favour the occurrence of severe anaphylactic reactions such as ACEi, beta blockers and thyroid therapeutics were also reported concomitantly in our datasets. However, this may also reflect their frequent use as these drug classes were also among the ten most frequently used drug classes in adults in Germany [35]. Thus, without considering drug prescription data on the individual patient level, we cannot evaluate if the reported co-medications were more frequently used by patients with anaphylactic reactions than in the general population. However, in the case of beta blockers and ACEi, their impact on mast cells, along with their blood pressure regulation, is discussed in the literature as possible influencing factors in this context [40–44].

Concerning the medical histories, pre-existing cardiovascular and respiratory diseases may impair compensatory mechanisms of the body (e.g. counter-regulation in the event of drop in blood pressure) and thus indirectly favour severe anaphylactic reactions [45]. In addition, infections, atopic and respiratory diseases (e.g. asthma) are described as risk factors in the literature [24, 29, 45, 46]. Regarding coronary artery diseases, the proportion of patients with coronary artery diseases (7.3%) in the spontaneous reports with information was slightly higher than expected in the German population (5.8%) [47]. Since one can assume an underreporting of medical histories in spontaneous reports, an even higher proportion can be expected. In the registry cases, only the summarizing term cardiovascular diseases was reported (36.7%). It is assumed that 8.0% and 6.1% of German adults suffer from asthma and COPD [47]. In the spontaneous reports the proportion for asthma (6.9%) and COPD (5.3%) was slightly lower, but in the registry cases the proportion of patients with asthma/COPD (20.8%) was clearly higher than expected in the general population. A prevalence of 3.8% was found for thyroid disorders and of 3.1% for hypothyroidism for Europe in a review analysis [48]. In our study, a clearly higher proportion of thyroid disorders was found in registry cases (16.7%) and a slightly higher proportion for hypothyroidism in the spontaneous reports (4.1%). The proportion of German adults with allergy (31.0%) in the general population [47] is clearly higher than the proportion in the spontaneous reports (23.1% (*n* = 434)). In a Bavarian study of rural areas, roughly 12% of females and roughly 6% of males reported an existing allergy against drugs [49]. However, only patients consulting doctors in rural areas who filled out a self-reporting questionnaire were considered. In 0.7% of the spontaneous reports in our study an already known hypersensitivity against the suspected drug was reported. The proportion (0.7%) was lower than in an Australian study (~ 6%), a German study of anaphylactic reactions in children (13.8%) and a pan-European study (18.5%) [5, 33, 50]. Nevertheless as these anaphylactic reactions may be avoidable, this finding emphasises the importance of the good medical practice, which requires physicians to ask the patients about known hypersensitivities and allergies when inquiring the medical history as part of the anamnesis or examine the patient records [51]. Additionally, patients should be encouraged to confirm their allergies by a specialised allergists and then document their allergies, e.g. in an allergy passport [52, 53]. In case of a pre-existing allergy and no alternative treatment options, appropriate precautions and pre-treatments have to be considered [54–58]. Furthermore, with careful attention to contraindications, desensitisation may offer a potential solution to allow the administration of the non-replaceable drug [59].

As a concluding remark, we can only compare our data to the prevalences published in the general population. We have no data regarding the distribution of diseases among patients taking specific drugs.

### Drug group and route of administration specific characteristics of anaphylactic reactions

Whether the drug group-specific associated conditions identified here (asthma ↔ analgesics; antibiotics ↔ infections; cardiovascular diseases ↔ contrast media) actually represent drug or drug group-specific risk factors, or if the more frequent occurrence can be explained by other factors (e.g. indication antibiotics: infections—potential confounder), needs to be investigated in further studies, whose methodology allows statements on causal inferences.

Atopy-associated conditions were more frequently present in reports of anaphylactic reactions related to orally administered drugs compared to those with intravenously administered drugs. A possible explanation could be that a history of atopy/allergy is ascertained with particular attention before intravenous administration of a drug, thus reducing the risk of severe reactions. Regarding the manifestation of the symptoms, anaphylactic reactions caused by intravenously administered drugs tended to affect the cardiovascular system and respiratory system more often. This may be due to the faster availability of intravenously administered drugs in the body or to differences among the patient populations (e.g. age and pre-existing conditions).

### Limitations

The incidence of anaphylactic reactions caused by certain drugs cannot be determined based on our data, since the data sources do not record all anaphylactic reactions (underreporting). Furthermore, we do not know precisely how often the respective drugs are used. Additionally, not all information (e.g. regarding the history of the patients) might be reported.

Furthermore, only a random sample of all spontaneous reports was analysed for the correctness of the diagnosis of the anaphylactic reaction. Thus, we cannot entirely rule out that hypersensitivity reactions (e.g. type IV hypersensitivity) other than anaphylactic reactions are included in our analyses of the spontaneous reports. In addition, we cannot rule out that some of the reports/case may be included in both datasets.

## Conclusion

Based on the findings derived from two different data sources, this study was able to show a more complete picture of severe drug-associated anaphylactic reactions in a real-world setting. Our results confirm that patients with thyroid disorders, cardiovascular and respiratory diseases (e.g. asthma) might carry a higher risk to develop severe anaphylactic reactions. Although the proportion was low, our study highlights the importance of enquiring about pre-existing drug hypersensitivities when taking the medical history of the patient, as these anaphylactic reactions may be avoidable. The associated factors identified to be reported more frequently for specific drug classes have to be investigated in other studies.

## Supplementary Information

Below is the link to the electronic supplementary material.

Supplementary file1 (DOCX 72.0 KB)

## Data Availability

The pseudonymised ADR reports from EudraVigilance are not publicly accessible due to data protection requirements. Distinct levels of access are provided for various stakeholders (https://www.ema.europa.eu/en/human-regulatory/research-development/pharmacovigilance/eudravigilance/access-eudravigilance-data). Being one of the competent authorities in Germany, the highest level of access is granted to the Federal Institute for Drugs and Medical Devices (BfArM). Nevertheless, even with lowest access level, researchers can perform the same analysis in EudraVigilance (EV) with aggregated data (public access: www.adrreports.eu/en/index.html). For further information regarding the processing of personal data in the context of the operation of EudraVigilance Human we refer to the European Medicines Agency’s Data Protection Notice for EudraVigilance Human. The pseudonymised reports from the Anaphylaxis Registry are not publicly accessible due to data protection requirements.

## References

[1] Lieberman P, Garvey LH (2016) Mast cells and anaphylaxis. Curr Allergy Asthma Rep 16(3):20. 10.1007/s11882-016-0598-5 26857018 10.1007/s11882-016-0598-5

[2] McNeil BD, Pundir P, Meeker S et al (2015) Identification of a mast-cell-specific receptor crucial for pseudo-allergic drug reactions. Nature 519(7542):237–241. 10.1038/nature14022 25517090 10.1038/nature14022 PMC4359082

[3] Johansson SGO, Bieber T, Dahl R et al (2004) Revised nomenclature for allergy for global use: report of the Nomenclature Review Committee of the World Allergy Organization, October 2003. Allergy Clin Immunol 113(5):832–836. 10.1016/j.jaci.2003.12.591 10.1016/j.jaci.2003.12.591 15131563

[4] Montañez MI, Mayorga C, Bogas G et al (2017) Epidemiology, mechanisms, and diagnosis of drug-induced anaphylaxis. Front Immunol 8:614. 10.3389/fimmu.2017.00614 28611774 10.3389/fimmu.2017.00614 PMC5446992

[5] Worm M, Moneret-Vautrin A, Scherer K et al (2014) First European data from the network of severe allergic reactions (NORA). Allergy 69(10):1397–1404. 10.1111/all.12475 24989080 10.1111/all.12475

[6] Demir S, Erdenen F, Gelincik A et al (2019) Evaluation of the potential risk factors for drug-induced anaphylaxis in adult patients. Int Arch Allergy Immunol 178(2):167–176. 10.1159/000494130 30448840 10.1159/000494130

[7] Hanschmann T, Francuzik W, Dölle-Bierke S et al (2023) Different phenotypes of drug-induced anaphylaxis—data from the European Anaphylaxis Registry. Allergy 78(6):1615–1627. 10.1111/all.15612 36479710 10.1111/all.15612

[8] Muraro A, Roberts G, Worm M et al (2014) Anaphylaxis: guidelines from the European Academy of Allergy and Clinical Immunology. Allergy 69(8):1026–1045. 10.1111/all.12437 24909803 10.1111/all.12437

[9] Francuzik W, Ruëff F, Bauer A et al (2021) Phenotype and risk factors of venom-induced anaphylaxis: a case-control study of the European Anaphylaxis Registry. J Allergy Clin Immunol 147(2):653-662.e9. 10.1016/j.jaci.2020.06.008 32585173 10.1016/j.jaci.2020.06.008

[10] Dölle-Bierke S, Höfer V, Francuzik W et al (2023) Food-induced anaphylaxis: data From the European Anaphylaxis Registry. J Allergy Clin Immunol Pract 11(7):2069-2079.e7. 10.1016/j.jaip.2023.03.026 36990430 10.1016/j.jaip.2023.03.026

[11] Höfer V, Dölle-Bierke S, Francuzik W et al (2024) Fatal and near-fatal anaphylaxis: data from the European Anaphylaxis Registry and National Health Statistics. J Allergy Clin Immunol Pract 12(1):96-105.e8. 10.1016/j.jaip.2023.09.044 37816460 10.1016/j.jaip.2023.09.044

[12] Tanno LK, Demoly P (2021) Epidemiology of anaphylaxis. Curr Opin Allergy Clin Immunol 21(2):168–174. 10.1097/ACI.0000000000000722 33560740 10.1097/ACI.0000000000000722

[13] European Medicines Agency (EMA). European medicines agency. Public data from Article 57 database. https://www.ema.europa.eu/en/human-regulatory/post-authorisation/data-medicines-iso-idmp-standards/public-data-article-57-database. Accessed 28 Mar 2024

[14] International Council for Harmonisation of, Technical Requirements for Pharmaceuticals for Human Use (ICH). Medical Dictionary for Regulatory Activities (MedDRA). https://www.meddra.org. Accessed 28 Mar 2024

[15] International Council for Harmonisation of Technical, Requirements for Pharmaceuticals for Human Use. Introductory Guide for Standardised MedDRA Queries (SMQs) Version 23.1. https://www.meddra.org/standardised-meddra-queries. Accessed 28 Mar 2024

[16] European Medicines Agency (EMA). Guideline on good pharmacovigilance practices (GVP) Module VI – collection, management and submission of reports of suspected adverse reactions to medicinal products (Rev 2). https://www.ema.europa.eu/en/documents/regulatory-procedural-guideline/guideline-good-pharmacovigilance-practices-gvp-module-vi-collection-management-submission-reports_en.pdf. Accessed 28 Mar 2024

[17] Dubrall D, Schmid M, Alešik E, Paeschke N, Stingl J, Sachs B (2018) Frequent adverse drug reactions, and medication groups under suspicion. Dtsch Arztebl Int 115(23):393–400. 10.3238/arztebl.2018.0393 29960607 10.3238/arztebl.2018.0393 PMC6041966

[18] Worm M, Eckermann O, Dölle S et al (2014) Triggers and Treatment of anaphylaxis: an analysis of 4,000 cases from Germany. Austria and Switzerland Dtsch Arztebl Int 111(21):367–375. 10.3238/arztebl.2014.0367 24939374 10.3238/arztebl.2014.0367 PMC4075276

[19] Ring J, Messmer K (1977) Incidence and severity of anaphylactoid reactions to colloid volume substitutes. Lancet 1(8009):466–469. 10.1016/S0140-6736(77)91953-5 65572 10.1016/s0140-6736(77)91953-5

[20] Edwards IR, Aronson JK (2000) Adverse drug reactions: definitions, diagnosis, and management. Lancet 356(9237):1255–1259. 10.1016/S0140-6736(00)02799-9 11072960 10.1016/S0140-6736(00)02799-9

[21] Landis JR, Koch GG (1977) The measurement of observer agreement for categorical data. Biometrics 33(1):159–174 843571

[22] Rothman KJ, Lanes S, Sacks ST (2004) The reporting odds ratio and its advantages over the proportional reporting ratio. Pharmacoepidemiol Drug Saf 13(8):519–523. 10.1002/pds.1001 15317031 10.1002/pds.1001

[23] Jerschow E, Lin RY, Scaperotti MM, McGinn AP (2014) Fatal anaphylaxis in the United States, 1999–2010: temporal patterns and demographic associations. J Allergy Clin Immunol 134(6):1318-1328.e7. 10.1016/j.jaci.2014.08.018 25280385 10.1016/j.jaci.2014.08.018 PMC4260987

[24] Turner PJ, Jerschow E, Umasunthar T, Lin R, Campbell DE, Boyle RJ (2017) Fatal anaphylaxis: mortality rate and risk factors. J Allergy Clin Immunol Pract 5(5):1169–1178. 10.1016/j.jaip.2017.06.031 28888247 10.1016/j.jaip.2017.06.031 PMC5589409

[25] Cardona V, Ansotegui IJ, Ebisawa M et al (2020) World allergy organization anaphylaxis guidance 2020. World Allergy Organ J 13(10):100472. 10.1016/j.waojou.2020.100472 33204386 10.1016/j.waojou.2020.100472 PMC7607509

[26] Hasford J, Goettler M, Munter KH, Müller-Oerlinghausen B (2002) Physicians’ knowledge and attitudes regarding the spontaneous reporting system for adverse drug reactions. J Clin Epidemiol 55(9):945–950. 10.1016/S0895-4356(02)00450-X 12393084 10.1016/s0895-4356(02)00450-x

[27] Christ P, Dubrall D, Schmid M, Sachs B. Comparative analysis of information provided in german adverse drug reaction reports sent by physicians, pharmacists and consumers. Drug Saf. 2023 Dec;46(12):1363–1379. 10.1007/s40264-023-01355-8. Epub 2023 Nov 21. PMID: 37987966; PMCID: PMC10684666. 10.1007/s40264-023-01355-8 PMC10684666 37987966

[28] De Germay S, Singier A, Salvo F, Pariente A; French pharmacovigilance network. Impact of Covid-19 vaccination on spontaneous pharmacovigilance reporting in France. Drug Saf (2023) 46(12):1381–1389. 10.1007/s40264-023-01359-4 37926785

[29] Yu RJ, Krantz MS, Phillips EJ, Stone CA (2021) Emerging causes of drug-induced anaphylaxis: a review of anaphylaxis-associated reports in the FDA adverse event reporting system (FAERS). J Allergy Clin Immunol Pract 9(2):819-829.e2. 10.1016/j.jaip.2020.09.021 32992044 10.1016/j.jaip.2020.09.021 PMC7870524

[30] Francuzik W, Kraft M, Hofmeier KS et al (2021) Anaphylaxis in middle-aged patients. Allergol Select 5(01):133–139. 10.5414/ALX02216E 33778366 10.5414/ALX02216E PMC7991893

[31] Warrington R, Silviu-Dan F, Wong T (2018) Drug allergy. *Allergy Asthma*. Clin Immunol 14(S2):60. 10.1186/s13223-018-0289-y 10.1186/s13223-018-0289-y PMC6157123 30275849

[32] Dhopeshwarkar N, Sheikh A, Doan R et al (2019) Drug-induced anaphylaxis documented in electronic health records. J Allergy Clin Immunol Pract 7(1):103–111. 10.1016/j.jaip.2018.06.010 29969686 10.1016/j.jaip.2018.06.010 PMC6311439

[33] Mullins RJ, Wainstein BK, Barnes EH, Liew WK, Campbell DE (2016) Increases in anaphylaxis fatalities in Australia from 1997 to 2013. Clin Exp Allergy 46(8):1099–1110. 10.1111/cea.12748 27144664 10.1111/cea.12748

[34] Sarganas G, Buttery AK, Zhuang W et al (2015) Prevalence, trends, patterns and associations of analgesic use in Germany. BMC Pharmacol Toxicol 16:28. 10.1186/s40360-015-0028-7 26428626 10.1186/s40360-015-0028-7 PMC4591581

[35] Knopf H, Grams D (2013) Results of the German Health Interview and Examination Survey for Adults (DEGS1). Bundesgesuntheitsbl 56:868–877. 10.1007/s00103-013-1667-8 10.1007/s00103-013-1667-8 23703508

[36] Cardona V, Guilarte M, Luengo O, Labrador-Horrillo M, Sala-Cunill A, Garriga T (2011) Allergic diseases in the elderly. Clin Transl Allergy 1(1):11. 10.1186/2045-7022-1-11 22409889 10.1186/2045-7022-1-11 PMC3339328

[37] Ludwig WD, Mühlbauer B, Seifert R, eds. *Arzneiverordnungs-Report 2022*. Springer Berlin Heidelberg; 2022. 10.1007/978-3-662-66303-5

[38] Hazell L, Shakir SAW (2006) Under-reporting of adverse drug reactions: a systematic review. Drug Saf 29(5):385–396. 10.2165/00002018-200629050-00003 16689555 10.2165/00002018-200629050-00003

[39] Sugizaki C, Sato S, Yanagida N, Ebisawa M (2023) Analysis of drug-induced anaphylaxis cases using the Japanese Adverse Drug Event Report (JADER) database – secondary publication. Allergol Int 72(4):580–587. 10.1016/j.alit.2023.03.006 37055270 10.1016/j.alit.2023.03.006

[40] Papadopoulos NG, Agache I, Bavbek S et al (2012) Research needs in allergy: an EAACI position paper, in collaboration with EFA. Clin Transl Allergy 2(1):21. 10.1186/2045-7022-2-21 23121771 10.1186/2045-7022-2-21 PMC3539924

[41] Sala-Cunill A, Cardona V (2021) Anaphylaxis viewed by experts: unmet needs. Curr Opin Allergy Clin Immunol 21(5):435–441. 10.1097/ACI.0000000000000771 34269744 10.1097/ACI.0000000000000771

[42] Wölbing F, Fischer J, Köberle M, Kaesler S, Biedermann T (2013) About the role and underlying mechanisms of cofactors in anaphylaxis. Allergy 68(9):1085–1092. 10.1111/all.12193 23909934 10.1111/all.12193

[43] Muñoz-Cano R, Pascal M, Araujo G et al (2017) Mechanisms, cofactors, and augmenting factors involved in anaphylaxis. Front Immunol 8:1193. 10.3389/fimmu.2017.01193 29018449 10.3389/fimmu.2017.01193 PMC5623009

[44] Nassiri M, Babina M, Dölle S, Edenharter G, Ruëff F, Worm M (2015) Ramipril and metoprolol intake aggravate human and murine anaphylaxis: evidence for direct mast cell priming. J Allergy Clin Immunol 135(2):491–499. 10.1016/j.jaci.2014.09.004 25441633 10.1016/j.jaci.2014.09.004

[45] Regateiro FS, Marques ML, Gomes ER (2020) Drug-induced anaphylaxis: an update on epidemiology and risk factors. Int Arch Allergy Immunol 181(7):481–487. 10.1159/000507445 32396909 10.1159/000507445

[46] Niggemann B, Beyer K (2014) Factors augmenting allergic reactions. Allergy 69(12):1582–1587. 10.1111/all.12532 25306896 10.1111/all.12532

[47] Robert Koch-Institut. Gesundheit in Deutschland aktuell – GEDA 2019/2020-EHIS. Tableau public. https://public.tableau.com/app/profile/robert.koch.institut/viz/Gesundheit_in_Deutschland_aktuell/GEDA_20192020-EHIS

[48] Madariaga AG, Santos Palacios S, Guillén-Grima F, Galofré JC (2014) The incidence and prevalence of thyroid dysfunction in Europe: a meta-analysis. J Clin Endocrinol Metab 99(3):923–931. 10.1210/jc.2013-2409 24423323 10.1210/jc.2013-2409

[49] Boehmer D, Schuster B, Krause J et al (2018) Prevalence and treatment of allergies in rural areas of Bavaria, Germany: a cross-sectional study. World Allergy Organ J 11:36. 10.1186/s40413-018-0218-z 30473740 10.1186/s40413-018-0218-z PMC6241034

[50] Sachs B, Dubrall D, Fischer-Barth W, Schmid M, Stingl J (2019) Drug-induced anaphylactic reactions in children: a retrospective analysis of 159 validated spontaneous reports. Pharmacoepidemiol Drug Saf 28(3):377–388. 10.1002/pds.4726 30706619 10.1002/pds.4726 PMC6590409

[51] Worm M, Francuzik W, Renaudin J-M et al (2018) Factors increasing the risk for a severe reaction in anaphylaxis: an analysis of data from the European Anaphylaxis Registry. Allergy 73(6):1322–1330. 10.1111/all.13380 29318637 10.1111/all.13380

[52] Brockow K, Aberer W, Atanaskovic-Markovic M et al (2016) Drug allergy passport and other documentation for patients with drug hypersensitivity - an ENDA/EAACI Drug Allergy Interest Group Position Paper. Allergy 71(11):1533–1539. 10.1111/all.12929 27145347 10.1111/all.12929

[53] Antolín-Amérigo D, Vidal-Albareda C, González de Olano D, de la Hoz-Caballer B (2024) Current update on anaphylaxis: anaphylaxis management in recent guidelines. Eur Ann Allergy Clin Immunol 56(2):51-64. https://doi.org/10.23822/EurAnnACI.1764-1489.306 10.23822/EurAnnACI.1764-1489.306 37462108

[54] Pagani M, Bavbek S, Alvarez-Cuesta E et al (2022) Hypersensitivity reactions to chemotherapy: an EAACI Position Paper. Allergy 77(2):388–403. 10.1111/all.15113 34587281 10.1111/all.15113

[55] Garvey LH (2016) Perioperative hypersensitivity reactions: diagnosis, treatment and evaluation. Curr Treat Options Allergy 3(2):113–128. 10.1007/s40521-016-0078-0

[56] Alvarez-Cuesta E, Madrigal-Burgaleta R, Broyles AD et al (2022) Standards for practical intravenous rapid drug desensitization & delabeling: a WAO committee statement. World Allergy Organ J 15(6):100640. 10.1016/j.waojou.2022.100640 35694005 10.1016/j.waojou.2022.100640 PMC9163606

[57] Muraro A, Worm M, Alviani C et al (2022) EAACI guidelines: anaphylaxis (2021 update). Allergy 77(2):357–377. 10.1111/all.15032 34343358 10.1111/all.15032

[58] Brockow K, Przybilla B, Aberer W et al (2015) Guideline for the diagnosis of drug hypersensitivity reactions: S2K-Guideline of the German Society for Allergology and Clinical Immunology (DGAKI) and the German Dermatological Society (DDG) in collaboration with the Association of German Allergologists (AeDA), the German Society for Pediatric Allergology and Environmental Medicine (GPA), the German Contact Dermatitis Research Group (DKG), the Swiss Society for Allergy and Immunology (SGAI), the Austrian Society for Allergology and Immunology (ÖGAI), the German Academy of Allergology and Environmental Medicine (DAAU), the German Center for Documentation of Severe Skin Reactions and the German Federal Institute for Drugs and Medical Products (BfArM). Allergo J Int 24(3):94–105. 10.1007/s40629-015-0052-6 26120552 10.1007/s40629-015-0052-6 PMC4479479

[59] Scherer K, Brockow K, Aberer W et al (2013) Desensitization in delayed drug hypersensitivity reactions – an EAACI position paper of the Drug Allergy Interest Group. Allergy 68(7):844–852. 10.1111/all.12161 23745779 10.1111/all.12161

