# Supplementary material for: Severe drug-associated anaphylaxis: a complementary descriptive analyses of registry cases and spontaneous reports
Source: Eur J Clin Pharmacol. 2025 Jun 27;81(9):1301–14. doi: 10.1007/s00228-025-03868-w (PMC12398439; doi:10.1007/s00228-025-03868-w)
Supplement: Supplementary file 1 — Supplementary file1 (DOCX 72.0 KB) [file 228_2025_3868_MOESM1_ESM.docx]

Supplementary Information (SI)

Title

**Severe drug-associated anaphylaxis: A complementary descriptive analyses of registry cases and spontaneous reports**

Name of the journal:

European Journal of Clinical Pharmacology

Authors

Dr. Patrick Christ ^1,2^; Dr. Diana Dubrall ^1,2^; Dr. rer. medic. Sabine Dölle-Bierke ^3^; Dr. med. Wojciech Francuzik ^3^; Prof. Dr. rer. nat. Matthias Schmid ^2^; Prof. Dr. med. Bernhardt Sachs ^1,4^; Prof. Dr. med. Margitta Worm ^3^

Margitta Worm and Bernhardt Sachs should be considered joint senior author

Author's institutional affiliations

1 Federal Institute for Drugs and Medical Devices (BfArM), Bonn

2 Institute for Medical Biometry, Informatics and Epidemiology (IMBIE), University Hospital of Bonn, Bonn, Germany

3 Division of Allergy and Immunology, Department of Dermatology, Venerology and Allergology, Charité—Universitätsmedizin Berlin, Corporate Member of Freie Universität Berlin, Humboldt-Universität zu Berlin, Berlin Institute of Health, Berlin, Germany

4 Department for Dermatology and Allergy, University Hospital RWTH Aachen, Aachen, Germany

Corresponding authors

| Prof. Dr. med. Bernhardt Sachs  [Bernhardt.Sachs@bfarm.de](mailto:Bernhardt.Sachs@bfarm.de)  0228/99 307 3156 | Prof. Dr. med. Margitta Worm  [margitta.worm@charite.de](https://email.charite.de/owa/UrlBlockedError.aspx)  030/ 450 518 092 |
| --- | --- |

SI Table 1. Descriptive analysis of spontaneous ADR reports from EudraVigilance.

| **Observation** | **EudraVigilance**  **[n=1,878]** |
| --- | --- |
| Reports per year | |
| Year | Number of reports  [%] |
| 2008 | 205 [10.9%] |
| 2009 | 179 [9.5%] |
| 2010 | 175 [9.3%] |
| 2011 | 148 [7.9%] |
| 2012 | 160 [8.5%] |
| 2013 | 127 [6.8%] |
| 2014 | 113 [6.0%] |
| 2015 | 122 [6.5%] |
| 2016 | 132 [7.0%] |
| 2017 | 106 [5.6%] |
| 2018 | 128 [6.8%] |
| 2019 | 107 [5.7%] |
| 2020 | 113 [6.0%] |
| 2021 | 63 [3.4%] |
| Total | 1,878 [100%] |
| Mean and median number of reports per year | |
| Median [interquartile range] | 127.5 [44.0 ] |
| Mean [standard deviation] | 134.1 [±36.5] |
| Age groups of the patients | |
| Age groups  [age in years] | Number of reports  [%] |
| Younger adults [18-44] | 542 [28.9%] |
| Older adults [45-64] | 732 [39.0%] |
| Seniors [≥ 65] | 604 [32.2%] |
| Mean and median age of the patients | |
| Median [interquartile range] | 55 [26] |
| Mean [standard deviation] | 54.4 [16.5] |
| Sex of the patients | |
| Sex | Number of reports  [%] |
| Female | 1,091 [58.1%] |
| Male | 777 [41.4%] |
| Not specified | 10 [0.5%] |
| Seriousness according to legal definition | |
| Classification of seriousness | Number of reports  [% on the total number of reports/ % on the total number of reports including any information] |
| Hospitalisation or prolongation thereof | |
| Hospitalisation | 529 [28.2%/ 56.6%] |
| No Hospitalisation | 405 [21.6%/ 43.4%] |
| Not specified | 944 [50.3%/ -] |
| Life-threatening | |
| Life-threatening | 821 [43.7%/ 55.4%] |
| Not Life-threatening | 661 [35.2%/ 44.6%] |
| Not specified | 396 [21.1%/ -] |
| Death of patient | |
| Patient died | 96 [5.1%/ 8.5%] |
| Patient alive | 1,031 [54.9%/ 91.5%] |
| Not specified | 751 [40.0%/ -] |

SI table 1 shows the number of spontaneous ADR reports per year, the demographical parameters of the patients and the seriousness criteria of the reports according to legal definitions.

SI Table 2. Descriptive analysis of associated factors, reported ADRs and suspected drugs in the spontaneous ADR reports from EudraVigilance.

|  | **EudraVigilance** | |
| --- | --- | --- |
| Associated factors [top 20] | | |
| **Rank** | **Medical histories of the patients [PT level of MedDRA terminology]** | **Number of reports**  **[% on the total number of reports/ % on the total number of reports describing any medical history]** |
|  | At least one medical history present | 1,416 [75.4%] |
| 1 | Hypertension | 296 [15.8%/ 20.9%] |
| 2 | Drug hypersensitivity | 125 [6.7%/ 8.8%] |
| 3 | Coronary artery disease | 103 [5.5%/ 7.3%] |
| 4 | Asthma | 98 [5.2%/ 6.9%] |
| 5 | Hypersensitivity | 91 [4.8%/ 6.4%] |
| 6 | Seasonal allergy | 83 [4.4%/ 5.9%] |
| 7 | Tobacco user | 82 [4.4%/ 5.8%] |
| 8 | Chronic obstructive pulmonary disease | 75 [4.0%/ 5.3%] |
| 9 | Type 2 diabetes mellitus | 71 [3.8%/ 5.0%] |
| 10 | Obesity | 68 [3.6%/ 4.8%] |
| 11 | Tobacco abuse | 62 [3.3%/ 4.4%] |
| 12 | Hypothyroidism | 58 [3.1%/ 4.1%] |
| 13 | Food allergy | 47 [2.5%/ 3.3%] |
| 14 | Atrial fibrillation | 46 [2.4%/ 3.2%] |
| 15 | Crohn's disease | 43 [2.3%/ 3.0%] |
| 16 | Diabetes mellitus | 42 [2.2%/ 3.0%] |
| 17 | Surgery | 39 [2.1%/ 2.8%] |
| 18 | Drug intolerance | 37 [2.0%/ 2.6%] |
| 19 | Hyperlipidaemia | 37 [2.0%/ 2.6%] |
| 20 | Renal failure | 37 [2.0%/ 2.6%] |
| Reported co-medication | | |
| Co-medication superior of drug classes of interest | | |
| **Rank** | **Co-medication superior of drug classes of interest** | **Number of reports [% on the total number of reports/ % on the total number of reports describing at least one co-medication]** |
| - | Co-medication present | 788 [42.0%] |
| 1 | Non-steroidal anti-inflammatory drugs (NSAIDs) | 147 [7.8%/ 18.7%] |
| 2 | Beta blocking agents | 129 [6.9%/ 16.4%] |
| 3 | Proton-pump inhibitors | 100 [5.3%/ 12.7%] |
| 4 | Thyroid therapeutics | 98 [5.2%/ 12.4%] |
| 5 | Angiotensin-converting enzyme (ACE) inhibitors | 95 [5.1%/ 12.1%] |
| 6 | Angiotensin II receptor antagonists | 78 [4.2%/ 9.9%] |
| 6 | Statins | 78 [4.2%/ 9.9%] |
| Top 10 co-medication (active ingredient) | | |
| **Rank** | **Co-medication (active ingredient)** | **Number of reports [% on the total number of reports/ % on the total number of reports describing at least one co-medication]** |
| - | Co-medication present | 788 [42.0%] |
| 1 | Levothyroxine | 95 [5.1%/ 12.1%] |
| 2 | Acetylsalicylic acid | 82 [4.4%/ 10.4%] |
| 3 | Prednisolone | 73 [3.9%/ 9.3%] |
| 4 | Dexamethasone | 63 [3.4%/ 8.0%] |
| 5 | Pantoprazole | 60 [3.2%/ 7.6%] |
| 6 | Metoprolol | 59 [3.1%/ 7.5%] |
| 7 | Ramipril | 57 [3.0%/ 7.2%] |
| 8 | Simvastatin | 54 [2.9%/ 6.9%] |
| 9 | Hydrochlorothiazid | 43 [2.3%/ 5.5%] |
| 10 | Bisoprolol | 42 [2.2%/ 5.3%] |
| Top 20 reported ADRs | | |
| **Rank** | **ADRs** | **Number of reports**  **[%]** |
| 1 | Anaphylactic shock | 1,068 [56.9%] |
| 2 | Anaphylactic reaction | 589 [31.4%] |
| 3 | Dyspnoea | 532 [28.3%] |
| 4 | Nausea | 223 [11.9%] |
| 5 | Urticaria | 215 [11.4%] |
| 6 | Pruritus | 205 [10.9%] |
| 7 | Circulatory collapse | 199 [10.6%] |
| 8 | Tachycardia | 198 [10.5%] |
| 9 | Hypotension | 197 [10.5%] |
| 10 | Erythema | 196 [10.4%] |
| 11 | Rash | 190 [10.1%] |
| 12 | Blood pressure decreased | 184 [9.8%] |
| 13 | Flushing | 147 [7.8%] |
| 14 | Dizziness | 121 [6.4%] |
| 15 | Bronchospasm | 118 [6.3%] |
| 16 | Loss of consciousness | 114 [6.1%] |
| 17 | Hypersensitivity | 110 [5.9%] |
| 18 | Angioedema | 99 [5.3%] |
| 19 | Vomiting | 97 [5.2%] |
| 20 | Chills | 91 [4.8%] |
| Type and number of affected organ systems | | |
| Type of affected organ systems | | |
| **Rank** | **Organ system considered according to Ring and Messmer ^19^** | **Number of reports**  **[% on the total number of reports]**  **/**  **Total number of reports describing at least one ADR affecting the respective organ system after exclusion of spontaneous reports only including the diagnoses anaphylactic/anaphylactoid reaction [% of subdataset]** |
| 1 | Skin symptoms | 852 [45.4%] / 588 [69.4%] |
| 2 | Cardiovascular system | 1,629 [86.7%] / 574 [67.8%] |
| 3 | Respiratory tract | 820 [43.7%] / 549 [64.8%] |
| 4 | Gastrointestinal tract | 463 [24.7%] / 336 [39.7%] |
| Number of affected organ systems | | |
|  | **Number of affected organ systems** **considered according to Ring and Messmer ^19^** | **Number of reports**  **[% on the total number of reports]**  **/**  **Total number of reports describing at least one ADR affecting the respective organ system after exclusion of spontaneous reports only including the diagnoses anaphylactic/anaphylactoid reaction [% of subdataset]** |
|  | One | 705 [37.5%] / 102 [13.2%] |
|  | Two | 597 [31.8%] / 322 [41.8%] |
|  | Three | 438 [23.3%] / 263 [34.1%] |
|  | Four | 138 [7.3%] / 84 [10.9%] |
| Reported suspected drugs | | |
| Suspected drugs superior of drug classes of interest | | |
|  | **Suspected drugs superior of drug classes of interest** | **Number of reports**  **[%]** |
|  | Antineoplastic agents | 381 [20.3%] |
|  | Antibiotics | 280 [14.9%] |
|  | Contrast media | 273 [14.5%] |
|  | Analgesics | 171 [9.1%] |
|  | Local anesthetics | 27 [1.4%] |
|  | Proton-pump inhibitors | 20 [1.1%] |
| Suspected drugs (active ingredients) | | |
| **Rank** | **Suspected drugs (active ingredients)** | **Number of reports**  **[%]** |
| **-** | ≥1 suspected drug per report | 416 [22.2%] |
| 1 | Moxifloxacin | 94 [5.0%] |
| 2 | Human immunoglobulins | 87 [4.6%] |
| 3 | Cefuroxime | 75 [4.0%] |
| 4 | Ferric carboxymaltose/Ferric polymaltose | 67 [3.6%] |
| 5 | Metamizole | 64 [3.4%] |
| 6 | Infliximab | 55 [2.9%] |
| 7 | Paclitaxel | 49 [2.6%] |
| 8 | Cetuximab | 43 [2.3%] |
| 9 | Iopromide | 42 [2.2%] |
| 10 | Iron/Iron dextran | 41 [2.2%] |
| 11 | Gadobutrol | 40 [2.1%] |
| 12 | Iomeprol | 39 [2.1%] |
| 13 | Gelatin-polysuccinat | 38 [2.0%] |
| 14 | Diclofenac | 37 [2.0%] |
| 15 | Carboplatin | 33 [1.8%] |
| 16 | Oxaliplatin | 33 [1.8%] |
| 17 | Glatiramer | 31 [1.7%] |
| 18 | Sulphur hexafluoride | 27 [1.4%] |
| 19 | Gadobenate | 23 [1.2%] |
| 20 | Gadoteridol | 21 [1.1%] |
| Route of application of suspected drugs | | |
| Top 10 routes of application | | |
| **Rank** | **Route of application** | **Number of reports describing at least one suspected drug administered via the respective route of application**  **[% on the total number of reports/ % on the total number of reports describing at least one route of application]** |
| - | No route of application specified | 446 [23.7%] |
| 1 | Intravenous | 918 [48.9%/64.9%] |
| 2 | Oral | 417 [22.2%/29.5%] |
| 3 | Subcutaneous | 114 [6.1%/8.1%] |
| 4 | Intramuscular | 34 [1.8%/2.4%] |
| 5 | Topical | 23 [1.2%/1.6%] |
| 6 | Cutaneous | 14 [0.7%/1.0%] |
| 7 | Respiratory (inhalation) | 14 [0.7%/1.0%] |
| 8 | Intra-arterial | 7 [0.4%/0.5%] |
| 9 | Ophthalmic | 6 [0.3%/0.4%] |
| 10 | Dental | 5 [0.3%/0.4%] |
| Reports describing exclusively orally or intravenously administered drugs | | |
|  | **Route of administration in report exclusively** | **Number of reports**  **[%]** |
|  | Oral | 371 [19.8%] |
|  | Intravenous | 811 [43.2%] |

ADR: adverse drug reaction

SI table 2 shows the medical histories of the patients, the ADRs and affected organ systems according to Ring and Messmer and the reported suspected and concomitants drugs.

SI Table 3. Analysis of spontaneous ADR reports from EudraVigilance with known allergy or hypersensitivity.

| Analysis of known allergies or hypersensitivities (n= 157) | | | |
| --- | --- | --- | --- |
|  | **Type of allergy or hypersensitivity** | **Number of reports**  **[% of the n= 157 reports]** | **Sum of reports per category [% of the n= 157 reports]** |
| **Unknown** allergy/hypersensitivity against suspected drug | Other elicitors | 55 [35.0%] | 143 [91.0%] |
|  | Other drugs | 36 [22.9%] |  |
|  | Other elicitors **&**  other drugs | 27 [17.2%] |  |
|  | No specific elicitor identified in the report | 25 [15.9%] |  |
| **Known** allergy/hypersensitivity against suspected drug | Suspected drug | 7 [4.5%] | 14 [9.0%] |
|  | Suspected drug **&**  other drugs | 3 [1.9%] |  |
|  | Suspected drug **&**  other elicitors | 2 [1.3%] |  |
|  | Suspected drug **&**  other elicitors **&**  other drugs | 2 [1.3%] |  |

SI Table 4. Characteristics more frequently reported in spontaneous reports of anaphylactic reactions to individual drug groups.

| **Drug Group Observed** | **Category Considered** | **Value in category** | **Number n of reports/cases**  **in observed drug group [Percentual share**  **of reports/cases in drug group observed]** | **Number n of reports/cases**  **in control drug group [Percentual share**  **of reports/cases in control group]** | **OR [CI+/-95%]** |
| --- | --- | --- | --- | --- | --- |
| **Analgesics** | ***Associated conditions*** | Asthma | 15 [8.8%] | 83 [4.9%] | 1.9 [1.1-3.3] |
|  |  | Hypersensitivity | 15 [8.8%] | 76 [4.5%] | 2.1 [1.2-3.7] |
|  | ***ADRs*** | Hypersensitivity | 20 [11.7%] | 90 [5.3%] | 2.4 [1.4-4.0] |
|  |  | Swollen tongue | 11 [6.4%] | 52 [3.0%] | 2.2 [1.1-4.3] |
| **Antibiotics** | ***Age group*** | Older adults 45-64 years | 136 [48.6%] | 596 [37.3%] | 1.6 [1.2-2.0] |
|  | ***Associated conditions*** | Asthma | 25 [8.9%] | 73 [4.6%] | 2.0 [1.3-3.3] |
|  |  | Bronchitis | 14 [5.0%] | 3 [0.2%] | 28.0 [8.0-98.0] |
|  |  | Chronic obstructive pulmonary disease | 22 [7.9%] | 53 [3.3%] | 2.5 [1.5-4.2] |
|  |  | Drug hypersensitivity | 30 [10.7%] | 95 [5.9%] | 1.9 [1.2-2.9] |
|  |  | Hypertension | 66 [23.6%] | 230 [14.4%] | 1.8 [1.3-2.5] |
|  |  | Obesity | 17 [6.1%] | 51 [3.2%] | 2.0 [1.1-3.4] |
|  |  | Tobacco abuse | 20 [7.1%] | 42 [2.6%] | 2.8 [1.6-4.9] |
|  |  | Tobacco user | 24 [8.6%] | 58 [3.6%] | 2.5 [1.5-4.1] |
|  | ***Co-medication*** | Ibuprofen | 11 [3.9%] | 25 [1.6%] | 2.6 [1.3-5.3] |
|  |  | Propofol | 13 [4.6%] | 26 [1.6%] | 2.9 [1.5-5.8] |
|  | ***ADRs*** | Anaphylactic shock | 178 [63.6%] | 890 [55.7%] | 1.4 [1.1-1.8] |
|  |  | Cardiovascular disorder | 19 [6.8%] | 59 [3.7%] | 1.9 [1.1-3.2] |
|  |  | Diarrhoea | 15 [5.4%] | 31 [1.9%] | 2.9 [1.5-5.4] |
|  |  | Erythema | 42 [15.0%] | 154 [9.6%] | 1.7 [1.1-2.4] |
|  |  | Pruritus | 42 [15.0%] | 163 [10.2%] | 1.6 [1.1-2.2] |
|  |  | Rash | 40 [14.3%] | 150 [9.4%] | 1.6 [1.1-2.3] |
|  |  | Resuscitation | 14 [5.0%] | 32 [2.0%] | 2.6 [1.4-4.9] |
|  |  | Tremor | 12 [4.3%] | 27 [1.7%] | 2.6 [1.3-5.2] |
|  | ***Number of affected organ systems*** | Four | 31 [11.1%] | 107 [6.7%] | 1.7 [1.1-2.6] |
|  | ***Affected organ systems*** | Skin | 160 [57.1%] | 692 [43.3%] | 1.7 [1.4-2.3] |
| **Antineplastic and immunomodulating agents** | ***Associated conditions*** | Chemotherapy | 13 [3.4%] | 22 [1.5%] | 2.4 [1.2-4.7] |
|  |  | Colitis ulcerosa | 12 [3.1%] | 8 [0.5%] | 6.1 [2.5-14.9] |
|  |  | Crohn’s disease | 26 [6.8%] | 17 [1.1%] | 6.4 [3.4-11.9] |
|  |  | Metastases to liver | 10 [2.6%] | 5 [0.3%] | 8.0 [2.7-23.7] |
|  |  | Multiple sclerosis | 25 [6.6%] | 9 [0.6%] | 11.6 [5.4-25.1] |
|  |  | Ovarian cancer | 13 [3.4%] | 4 [0.3%] | 13.2 [4.3-40.7] |
|  |  | Psoriasis | 10 [2.6%] | 6 [0.4%] | 6.7 [2.4-18.5] |
|  |  | Radiotherapy | 11 [2.9%] | 14 [0.9%] | 3.1 [1.4-7.0] |
|  |  | Rheumatoid arthritis | 12 [3.1%] | 9 [0.6%] | 5.4 [2.2-12.9] |
|  | ***Co-medication*** | Clemastine | 21 [5.5%] | 15 [1.0%] | 5.8 [2.9-11.3] |
|  |  | Dexamethasone | 42 [11.0%] | 18 [1.2%] | 10.2 [5.8-17.9] |
|  |  | Dimetindenee | 13 [3.4%] | 24 [1.6%] | 2.2 [1.1-4.3] |
|  |  | Granisetron | 15 [3.9%] | 3 [0.2%] | 20.4 [5.9-70.9] |
|  |  | Ondansetron | 15 [3.9%] | 2 [0.1%] | 30.6 [7.0-134.6] |
|  |  | Prednisolone | 28 [7.3%] | 45 [3.0%] | 2.6 [1.6-4.2] |
|  |  | Ranitidine | 21 [5.5%] | 19 [1.3%] | 4.5 [2.4-8.5] |
|  | ***ADRs*** | Cyanosis | 14 [3.7%] | 22 [1.5%] | 2.6 [1.3-5.0] |
|  | ***Number of affected organ systems*** | One | 193 [50.7%] | 511 [34.1%] | 2.0 [1.6-2.5] |
| **Contrast media** | ***Sex*** | Male | 135 [49.5%] | 642 [40.0%] | 1.5 [1.1-1.9] |
|  | ***Associated conditions*** | Coronary artery disease | 26 [9.5%] | 77 [4.8%] | 2.1 [1.3-3.3] |
|  | ***Co-medication*** | Cimetidine | 12 [4.4%] | 3 [0.2%] | 24.6 [6.9-87.6] |
|  |  | Cortisone | 10 [3.7%] | 14 [0.9%] | 4.3 [1.9-9.8] |
|  |  | Dimetindene | 15 [5.5%] | 22 [1.4%] | 4.2 [2.1-8.2] |
|  |  | Epinephrine | 17 [6.2%] | 7 [0.4%] | 15.2 [6.2-36.9] |
|  |  | Oxygen | 10 [3.7%] | 2 [0.1%] | 30.5 [6.6-139.9] |
|  | ***ADRs*** | Anaphylactoid reaction | 41 [15.0%] | 48 [3.0%] | 5.7 [3.7-8.9] |
|  |  | Anaphylactoid shock | 22 [8.1%] | 16 [1.0%] | 8.7 [4.5-16.8] |
|  |  | Cardiac arrest | 23 [8.4%] | 53 [3.3%] | 2.7 [1.6-4.5] |
|  |  | Malaise | 12 [4.4%] | 31 [1.9%] | 2.3 [1.2-4.6] |
|  |  | Nausea | 59 [21.6%] | 164 [10.2%] | 2.4 [1.7-3.4] |
|  |  | Seizure | 11 [4.0%] | 17 [1.1%] | 3.9 [1.8-8.5] |
|  |  | Sneezing | 12 [4.4%] | 4 [0.2%] | 18.4 [5.9-57.5] |
|  | ***Hospitalisation*** | Hospitalisation or prolongation thereof | 181 [66.3%] | 763 [47.5%] | 2.2 [1.7-2.8] |
| **Local anaesthetics** | ***Sex*** | Female | 22 [81.5%] | 1069 [57.8%] | 3.2 [1.2-8.5] |
|  | ***ADRs*** | Anaphylactic shock | 21 [77.8%] | 1047 [56.6%] | 2.7 [1.1-6.7] |
|  | ***Number of affected organ systems*** | One | 16 [59.3%] | 688 [37.2%] | 2.5 [1.1-5.3] |
| **PPI*** | ***Age group*** | Older adults 45-64 years | 13 [65.0%] | 719 [38.7%] | 2.9 [1.2-7.4] |

PPI: Proton pump inhibitor

SI table 4 shows the characteristics more frequently reported in spontaneous reports of anaphylactic reactions to the individual drug group compared to all reports of anaphylactic reactions to the other drug groups and their reporting odds ratios with their associated ±95% confidence intervals. A more frequent occurrence in the reports describing anaphylactic reactions to the individual drug group was assumed if the lower CI was greater than 1.0. The drug groups were compared with regard to the categories of sex and age group of the patients, medical histories, associated factors, co-medications, number and type of affected organ systems and the criterion of hospitalization. To restrict the analysis to the most important categories, at least 10 reports had to be present for the respective specification.

SI table 5. Analysis of spontaneous ADR reports exclusively including orally or intravenously administered drugs.

| **Category** | **Sub-category** | **Number of reports describing exclusively orally**  **administered drugs [% of the n= 371 reports exclusively including orally administered drugs]** | **Number n of reports describing exclusively intravenously**  **administered drugs**  **[% of the n= 811 reports exclusively including intravenously administered drugs]** | **OR  [CI ±95%]** |
| --- | --- | --- | --- | --- |
| **Age-groups** | Older adults [45-64] | 179 [48.2%] | 301 [37.1%] | 1.6 [1.2 - 2.0] |
|  | Seniors [≥ 65] | 87 [23.5%] | 309 [38.1%] | 0.5 [0.4 - 0.7] |
| **Associated conditions** | Asthma | 29 [7.8%] | 33 [4.1%] | 2.0 [1.2 - 3.3] |
|  | No medical history reported | 100 [27.0%] | 165 [20.3%] | 1.4 [1.1 - 1.9] |
|  | Food allergy | 19 [5.1%] | 7 [0.9%] | 6.2 [2.6 - 14.9] |
|  | Seasonal allergy | 23 [6.2%] | 26 [3.2%] | 2.0 [1.1 - 3.5] |
|  | Hypersensitivity | 23 [6.2%] | 27 [3.3%] | 1.9 [1.1 - 3.4] |
|  | Chemotherapy | 1 [0.3%] | 18 [2.2%] | 0.1 [<0.1 - 0.9] |
|  | Crohn’s disease | 1 [0.3%] | 26 [3.2%] | 0.1 [<0.1 - 0.6] |
|  | Renal failure | 3 [0.8%] | 29 [3.6%] | 0.2 [0.1 - 0.7] |
| **Death** | No death | 235 [63.3%] | 384 [47.3%] | 1.9 [1.5 - 2.5] |
|  | Death | 7 [1.9%] | 50 [6.2%] | 0.3 [0.1 - 0.7] |
| **ADRs** | Anaphylactic shock | 227 [61.2%] | 430 [53.0%] | 1.4 [1.1 - 1.8] |
|  | Angioedema | 41 [11.1%] | 23 [2.8%] | 4.3 [2.5 - 7.2] |
|  | Lip swelling | 25 [6.7%] | 11 [1.4%] | 5.3 [2.6 - 10.8] |
|  | Dyspnoea | 122 [32.9%] | 207 [25.5%] | 1.4 [1.1 - 1.9] |
|  | Respiratory distress | 21 [5.7%] | 21 [2.6%] | 2.3 [1.2 - 4.2] |
|  | Rash | 56 [15.1%] | 60 [7.4%] | 2.2 [1.5 - 3.3] |
|  | Diarrhoea | 22 [5.9%] | 9 [1.1%] | 5.6 [2.6 - 12.3] |
|  | Dysphagia | 17 [4.6%] | 6 [0.7%] | 6.4 [2.5 - 16.5] |
|  | Pruritus | 66 [17.8%] | 54 [6.7%] | 3.0 [2.1 - 4.5] |
|  | Swollen tongue | 29 [7.8%] | 11 [1.4%] | 6.2 [3.0 - 12.5] |
|  | Swollen face | 29 [7.8%] | 16 [2.0%] | 4.2 [2.3 - 7.9] |
|  | Dizziness | 38 [10.2%] | 36 [4.4%] | 2.5 [1.5 - 3.9] |
|  | Tremor | 15 [4%] | 12 [1.5%] | 2.8 [1.3 - 6.1] |
|  | Hypersensitivity | 25 [6.7%] | 31 [3.8%] | 1.8 [1.1 - 3.1] |
|  | Urticaria | 56 [15.1%] | 60 [7.4%] | 2.2 [1.5 - 3.3] |
|  | Anaphylactoid reaction | 6 [1.6%] | 49 [6.0%] | 0.3 [0.1 - 0.6] |
|  | Anaphylactoid shock | 1 [0.3%] | 24 [3.0%] | 0.1 [<0.1 - 0.7] |
|  | Hypotension | 29 [7.8%] | 108 [13.3%] | 0.6 [0.4 - 0.8] |
|  | Bronchospasm | 15 [4.0%] | 72 [8.9%] | 0.4 [0.2 - 0.8] |
|  | Cardiac arrest | 4 [1.1%] | 44 [5.4%] | 0.2 [0.1 - 0.5] |
|  | Oxygen saturation decreased | 3 [0.8%] | 23 [2.8%] | 0.3 [0.1 - 0.9] |
|  | Tachycardia | 31 [8.4%] | 105 [12.9%] | 0.6 [0.4 - 0.9] |
|  | Resuscitation | 3 [0.8%] | 33 [4.1%] | 0.2 [0.1 - 0.6] |

OR: odds ratio, CI: confidence intervals, ADR: adverse drug reaction

SI table 4 shows the characteristics more frequently reported in reports of exclusively orally or intravenously applied drugs and their reporting odds ratios with their associated ± 95% confidence intervals. A more frequent occurrence in reports with exclusively orally applied drugs was assumed if the lower CI was greater than 1.0 and a more frequent occurrence in reports with exclusively intravenously applied drugs was assumed if the upper CI was lower than 1.0. The blue lines present the characteristics more frequently reported in reports with exclusively orally applied drugs. The red lines present the characteristics more frequently reported in reports with exclusively intravenously applied drugs.

SI table 6. Descriptive analysis of registry cases from the anaphylaxis registry.

| **Observation** | **Anaphylaxis Registry**  **[n=1,046]** |
| --- | --- |
| Number of cases per year | |
| Year | Number of cases  [%] |
| 2008 | 47 [4.5%] |
| 2009 | 53 [5.1%] |
| 2010 | 76 [7.3%] |
| 2011 | 38 [3.6%] |
| 2012 | 86 [8.2%] |
| 2013 | 76 [7.3%] |
| 2014 | 287 [27.4%] |
| 2015 | 59 [5.6%] |
| 2016 | 55 [5.3%] |
| 2017 | 50 [4.8%] |
| 2018 | 67 [6.4%] |
| 2019 | 55 [5.3%] |
| 2020 | 46 [4.4%] |
| 2021 | 51 [4.9%] |
| Total | 1,046 [100%] |
| Mean and median number of cases per year | |
| Median [interquartile range] | 55 [23.5] |
| Mean [standard deviation] | 74.7 [±62.6] |
| Age groups of the patients | |
| Age groups  [age in years] | Number of cases  [%] |
| Younger adults [18-44] | 402 [38.4%] |
| Older adults [45-64] | 476 [45.5%] |
| Seniors [≥ 65] | 168 [16.1%] |
| Mean and median age of the patients | |
| Median [interquartile range] | 49 [23] |
| Mean [standard deviation] | 48.9 [15.5] |
| Sex of the patients | |
| Sex | Number of cases  [%] |
| Female | 725 [69.3%] |
| Male | 321 [30.7%] |
| Not specified | - |
| Severity of the cases | |
| Value of severity | Number of cases  [% on the total number of cases/ % on the total number of cases including any information] |
| Hospitalisation | |
| Hospitalisation | 193 [18.5%/ 26.9%] |
| No Hospitalisation | 524 [50.1%/ 73.1%] |
| Not specified | 329 [31.5%/ -] |
| Death of patient | |
| Patient died | 10 [1.0%] |
| Patient alive | 1,036 [99.0%] |
| Not specified | - |

SI table 6 shows the number of spontaneous ADR reports per year, the demographical parameters of the patients and the severity of the anaphylactic reactions.

SI table 7. Descriptive analysis of associated factors, reports ADRs and suspected drugs in the registry cases.

| **Anaphylaxis Registry** | | |
| --- | --- | --- |
| Associated factors [top 20] | | |
| **Rank** | **Associated factors [according to coding in the Anaphylaxis Registry]** | **Number of cases**  **[% on the total number of cases/ % on the total number of cases describing at least one associated factor]** |
|  | At least one associated factors present | 830 [79.3%] |
| 1 | Cardiovascular diseases | 305 [29.2%/ 36.7%] |
| 2 | Others | 285 [27.2%/ 34.3%] |
| 3 | Asthma/COPD | 173 [16.5%/ 20.8%] |
| 4 | Allergic rhinitis/ rhinoconjunctivitis | 165 [15.8%/ 19.9%] |
| 5 | Thyroid disease | 139 [13.3%/ 16.7%] |
| 6 | Stress likely | 138 [13.2%/ 16.6%] |
| 7 | Exercise | 129 [12.3%/ 15.5%] |
| 8 | Chronic infection | 73 [7.0%/ 8.8%] |
| 9 | Sleep deprivation | 56 [5.4%/ 6.7%] |
| 10 | Diabetes mellitus | 47 [4.5%/ 5.7%] |
| 11 | Atopic dermatitis/ eczema | 42 [4.0%/ 5.1%] |
| 12 | Malignant diseases | 36 [3.4%/ 4.3%] |
| 13 | Chronic urticaria | 35 [3.3%/ 4.2%] |
| 14 | Menopause | 30 [2.9%/ 3.6%] |
| 15 | Food allergies | 26 [2.5%/ 3.1%] |
| 16 | Polyposis nasi | 24 [2.3%/ 2.9%] |
| 17 | Menstruation | 22 [2.1%/ 2.7%] |
| 18 | Alcohol | 14 [1.3%/ 1.7%] |
| 19 | Mastocytosis | 10 [1.0%/ 1.2%] |
| 20 | - | - |
| Reported co-medication | | |
| Reported co-medication superior (selected drug groups) | | |
| **Rank** | **Co-medication superior of drug classes of interest** | **Number of cases**  **[% on the total number of cases/ % on the total number of cases describing at least one co-medication]** |
| - | Co-medication present | 534 [51.1%] |
| 1 | Beta blocking agents | 134 [12.8%/ 25.1%] |
| 2 | Thyroid therapeutics | 112 [10.7%/ 21.0%] |
| 3 | Angiotensin-converting enzyme (ACE) inhibitors | 92 [8.8%/ 17.2%] |
| 4 | Proton-pump inhibitors | 77 [7.4%/ 14.4%] |
| 5 | Angiotensin II receptor antagonists | 66 [6.3%/ 12.4%] |
| 6 | Diuretics | 60 [5.7%/ 11.2%] |
| 7 | Acetylsalicylic acid (low dose) | 59 [5.6%/ 11.0%] |
| 8 | Statins | 54 [5.2%/ 10.1%] |
| 9 | Calcium channel blockers | 50 [4.8%/ 9.4%] |
| 10 | Hormonal contraceptives | 23 [2.2%/ 4.3%] |
|  | Other drugs not categorised in groups | 321 [30.7%/ 60.1%] |
| Top 20 ADRs | | |
| **Rank** | **ADRs** | **Number of cases**  **[%]** |
| 1 | Dyspnea | 625 [59.8%] |
| 2 | Angioedema | 416 [39.8%] |
| 3 | Hypotension/collapse | 389 [37.2%] |
| 4 | Urticaria | 371 [35.5%] |
| 5 | Pruritus | 370 [35.4%] |
| 6 | Erythema/Flush | 341 [32.6%] |
| 7 | Dizziness | 264 [25.2%] |
| 8 | Loss of consciousness | 210 [20.1%] |
| 9 | Nausea | 201 [19.2%] |
| 10 | Tachycardia | 200 [19.1%] |
| 11 | Hot sweat/tremble | 156 [14.9%] |
| 12 | Throat tightness | 134 [12.8%] |
| 13 | Paraesthesia | 113 [10.8%] |
| 14 | Chest tightness | 103 [9.8%] |
| 15 | Vomiting | 102 [9.8%] |
| 16 | Reduction of alertness | 92 [8.8%] |
| 17 | Cardiac arrest | 63 [6.0%] |
| 18 | Diarrhoea | 61 [5.8%] |
| 19 | Palpitation | 60 [5.7%] |
| 20 | Stridor inspiratory | 54 [5.2%] |
| Type and number of affected organ systems | | |
| Type of affected organ systems | | |
| **Rank** | **Organ system considered according to Ring and Messmer ^19^** | **Number of cases describing**  **[%]** |
| 1 | Skin symptoms | 804 [76.9%] |
| 2 | Cardiovascular system | 749 [71.6%] |
| 3 | Respiratory tract | 745 [71.2%] |
| 4 | Gastrointestinal tract | 312 [29.8%] |
| Number of affected organ systems | | |
|  | **Number of affected organ systems** **considered according to Ring and Messmer ^19^** | **Number of cases**  **[%]** |
|  | One | 66 [6.3%] |
|  | Two | 513 [49%] |
|  | Three | 350 [33.5%] |
|  | Four | 117 [11.2%] |
| Reported suspected drugs | | |
| Suspected drugs superior (selected drug groups) | | |
| **Rank** | **Suspected drugs superior (selected drug groups)** | **Number of cases**  **[%]** |
| 1 | Analgesics | 442 [42.3%] |
| 2 | Antibiotics | 263 [25.1%] |
| 3 | Local anesthetics | 141 [13.5%] |
| 4 | Contrast media | 39 [3.7%] |
| 5 | Narcotics | 39 [3.7%] |
| 6 | Proton-pump inhibitors | 29 [2.8%] |
| 7 | Biologics | 10 [1.0%] |
| 8 | Glucocorticoids | 10 [1.0%] |
| 9 | Muscle relaxants | 10 [1.0%] |
| 10 | Additives | 8 [0.8%] |
| 11 | Cardiovascular drugs | 8 [0.8%] |
| 12 | Antineoplastic agents | 5 [0.5%] |
| 13 | Volume replacement (colloid solutions) | 3 [0.3%] |
| Reported suspected drugs (more detailed) | | |
| **Rank** | **Suspected drugs (more detailed)** | **Number of cases**  **[%]** |
| 1 | Metamizole | 123 [11.8%] |
| 2 | Cephalosporin | 109 [10.4%] |
| 3 | Diclofenac | 98 [9.4%] |
| 4 | Ibuprofen | 94 [9.0%] |
| 5 | Aspirin (Acetylsalicylic acid) | 75 [7.2%] |
| 6 | Penicillin | 63 [6.0%] |
| 7 | Quinolones (and other gyrase inhibitors) | 46 [4.4%] |
| 8 | Analgesics (not further specified) | 42 [4.0%] |
| 9 | X-ray contrast media (not further specified) | 29 [2.8%] |
| 10 | Narcotics (not further specified) | 27 [2.6%] |
| 11 | Proton pump inhibitors (not further specified) | 24 [2.3%] |
| 12 | Macrolides (e.g.erythromycin), Lincosamides (e.g.clindamycin), Streptogramins | 22 [2.1%] |
| 13 | Sulphonamides | 13 [1.2%] |
| 14 | Local anaesthetics (not further specified) | 12 [1.1%] |
| 15 | Paracetamol | 11 [1.1%] |
| 16 | TNF-alpha inhibitors | 4 [0.4%] |
| 17 | Antibiotics (not further specified) | 4 [0.4%] |
| 18 | Anesthetics (not further specified) | 4 [0.4%] |
| 19 | Celecoxib | 3 [0.3%] |
| 20 | Chlorhexidin | 3 [0.3%] |

ADR: adverse drug reaction

SI table 7 shows the associated conditions of the patients, the ADRs and affected organ systems according to Ring and Messmer and the reported suspected and concomitants drugs.

SI table 8. Characteristics more frequently reported in registry cases of anaphylactic reactions to individual drug groups.

| **Drug Group Observed** | **Category Considered** | **Value in category** | **Number n of reports/cases**  **in observed drug group [Percentual share**  **of reports/cases in drug group observed]** | **Number n of reports/cases**  **in control drug group [Percentual share**  **of reports/cases in control group]** | **OR [CI+/-95%]** |
| --- | --- | --- | --- | --- | --- |
| Analgesics | Sex | Male | 155 [36.7%] | 166 [26.6%] | 1.6 [1.2-2.1] |
|  | Associated conditions | Male | 155 [36.7%] | 166 [26.6%] | 1.6 [1.2-2.1] |
|  |  | Asthma | 86 [20.4%] | 87 [13.9%] | 1.6 [1.1-2.2] |
|  |  | Polyposis | 19 [4.5%] | 5 [0.8%] | 5.8 [2.2-15.8] |
|  |  | Chronic urticaria | 22 [5.2%] | 13 [2.1%] | 2.6 [1.3-5.2] |
|  |  | Exercise mild | 75 [17.8%] | 54 [8.7%] | 2.3 [1.6-3.3] |
|  |  | Exercise moderate | 51 [12.1%] | 34 [5.4%] | 2.4 [1.5-3.8] |
|  | ADRs | Angioedema | 202 [47.9%] | 214 [34.3%] | 1.8 [1.4-2.3] |
|  |  | Chest tightness | 52 [12.3%] | 51 [8.2%] | 1.6 [1.1-2.4] |
|  |  | Diarrhoea | 35 [8.3%] | 26 [4.2%] | 2.1 [1.2-3.5] |
|  |  | Dysphonia | 13 [3.1%] | 7 [1.1%] | 2.8 [1.1-7.1] |
|  |  | Pruritus | 190 [45.0%] | 180 [28.8%] | 2.0 [1.6-2.6] |
|  |  | Rhinitis | 20 [4.7%] | 12 [1.9%] | 2.5 [1.2-5.2] |
|  |  | Urticaria | 175 [41.5%] | 196 [31.4%] | 1.5 [1.2-2.0] |
|  | Affected organ systems | Skin | 351 [83.2%] | 453 [72.6%] | 1.9 [1.4-2.5] |
| Antibiotics | Sex | Female | 192 [77.1%] | 533 [66.9%] | 1.7 [1.2-2.3] |
|  | Associated conditions | Infection | 45 [18.1%] | 28 [3.5%] | 6.1 [3.7-10.0] |
|  | Co-medication | Hormonal contraceptives | 10 [4.0%] | 13 [1.6%] | 2.5 [1.1-5.8] |
| Contrast media | Agegroup | Seniors 65 years and older | 11 [29.7%] | 157 [15.6%] | 2.3 [1.1-4.7] |
|  | Associated conditions | Cardiovascular disease | 18 [48.6%] | 287 [28.4%] | 2.4 [1.2-4.6] |
| Localanaesthtics | Associated conditions | No associated conditions specified | 40 [32.5%] | 176 [19.1%] | 2.0 [1.4-3.1] |
|  |  | Stress likely | 26 [21.1%] | 112 [12.1%] | 1.9 [1.2-3.1] |
|  | ADRs | Hot sweat tremble | 34 [27.6%] | 122 [13.2%] | 2.5 [1.6-3.9] |
|  |  | Loss of consciousness | 35 [28.5%] | 175 [19.0%] | 1.7 [1.1-2.6] |
|  |  | Nausea | 35 [28.5%] | 166 [18.0%] | 1.8 [1.2-2.8] |
|  |  | Palpitation | 16 [13.0%] | 44 [4.8%] | 3.0 [1.6-5.5] |
|  | Number of affected organ systems | One | 21 [17.1%] | 45 [4.9%] | 4.0 [2.3-7.0] |
|  | Affected organ systems | Cardiovascular system | 107 [87.0%] | 642 [69.6%] | 2.9 [1.7-5.0] |
| PPI* | Reactions | Pruritus | 17 [63.0%] | 353 [34.6%] | 3.2 [1.5-7.1] |

PPI: Proton pump inhibitor

SI table 8 shows the characteristics more frequently reported in registry cases of anaphylactic reactions to the individual drug group compared to all cases of anaphylactic reactions to the other drug groups and their reporting odds ratios with their associated ±95% confidence intervals. A more frequent occurrence in the cases describing anaphylactic reactions to the individual drug group was assumed if the lower CI was greater than 1.0. The drug groups were compared with regard to the categories of sex and age group of the patients, medical histories, associated factors, co-medications, number and type of affected organ systems and the criterion of hospitalization. To restrict the analysis to the most important categories, at least 10 reports had to be present for the respective specification.
